# Non-Coding DNA-Derived Mimotopes of Aβ₄₂ as Novel Candidates for Alzheimer's Peptide Vaccine Design

**DOI:** 10.7150/ijms.127358

**Published:** 2026-03-04

**Authors:** Navya Raj, Shidhi P. R, Rebai Ben Ammar, Peramaiyan Rajendran, Biju Vadakkemukadyil Chellappan

**Affiliations:** 1 Department of Health Informatics, College of Health Sciences, Saudi Electronic University, Dammam, Saudi Arabia.; 2 Department of Computational Biology and Bioinformatics, University of Kerala, Thiruvananthapuram, Kerala, India; Department of Zoology, University of Kerala, Thiruvananthapuram, Kerala, India.; 3 Department of Biological Science, College of Science, King Faisal University, Al-Ahsa, 31982, Saudi Arabia.

**Keywords:** Alzheimer's disease (AD), Amyloid beta (Aβ) mimotopes, Epitope-based vaccine design, Immunoinformatics, Peptide-antibody docking, Noncoding DNA

## Abstract

Alzheimer's disease (AD), the leading cause of dementia, is a progressive neurodegenerative disorder marked by memory loss, cognitive decline, and characteristic neuropathology involving amyloid-β (Aβ) plaques and tau tangles. Among emerging therapeutic strategies, Aβ-targeted immunotherapy using monoclonal antibodies or peptide vaccines offers the most promising disease-modifying potential. Mimotopes, short peptides that mimic antigenic epitopes of Aβ, have recently gained attention as safe and effective candidates for vaccine development. In this study, we employed a computational immunoinformatics approach to identify novel Aβ_42_-mimicking peptides derived from non-coding DNA sequences, representing an unconventional yet rich source of bioactive peptides. A virtual peptide library was generated from intergenic regions of the *Escherichia coli* genome and screened using B-cell epitope prediction, MHC-binding analysis, and structural similarity modeling to identify potential immunogenic mimotopes. Selected candidates were further evaluated through peptide-antibody docking with Aβ_42_-specific antibody fragments to assess binding affinity and epitope mimicry. Our findings demonstrate a novel computational framework for mining non-coding DNA to identify therapeutic peptide mimotopes. The identified Aβ_42_-like peptides exhibit strong potential as synthetic vaccine candidates for Alzheimer's disease, supporting a new direction in rational vaccine design against neurodegenerative disorders.

## Introduction

Peptide-based vaccines have gained significant traction in recent years due to their low production cost, stability, and ease of handling, although their primary limitation remains low immunogenicity 1-3. Recent advances in immunoinformatics and reverse vaccinology now allow genome-scale identification of antigenic epitopes, enabling rational design of epitope-based vaccines with improved specificity and safety 4-7. Epitope-based vaccine design focuses on identifying short B- and T-cell epitopes capable of eliciting targeted immune responses while minimizing off-target effects. B-cell epitopes can be linear or conformational, whereas T-cell epitopes are presented by MHC molecules to activate T lymphocytes.

Mimotopes are short peptides that structurally or functionally mimic antigenic epitopes and can induce antibody responses similar to those raised against the native antigen 8-9. Because mimotopes reproduce the three-dimensional antibody-binding surface without requiring sequence identity, they offer a safe strategy when the native antigen is toxic, unstable, or poorly immunogenic 10-12. This property makes mimotopes especially attractive for diseases such as Alzheimer's disease (AD), where the target antigen itself is pathogenic 13.

Alzheimer's disease is a progressive neurodegenerative disorder that accounts for approximately 60-70% of dementia cases worldwide 14-15. According to the amyloid cascade hypothesis, abnormal processing of amyloid precursor protein generates amyloid-β (Aβ), particularly the 42-residue form (Aβ_42_), which aggregates into toxic oligomers and fibrils that drive synaptic dysfunction and neurodegeneration 16-17. Consequently, Aβ_42_ has been a central target of immunotherapy.

The first active immunization trial using full-length Aβ_42_ (AN-1792) successfully induced antibodies but caused T-cell-mediated meningoencephalitis in a subset of patients, leading to termination of the trial 18-21. Subsequent efforts therefore, shifted toward epitope-based vaccines that selectively target pathogenic Aβ conformers while avoiding autoimmune T-cell activation 22. Ghochikyan 23 proposed that mimotopes of toxic Aβ conformers could provide a safer and more effective vaccination strategy.

In parallel with advances in AD immunotherapy, it has become evident that genomic regions traditionally classified as non-coding harbor substantial latent coding potential 24-27. Short open reading frames within intergenic and pseudogene regions can give rise to stable, bioactive peptides 28-30. Building on this concept, our group previously demonstrated that artificial translation of bacterial non-coding regions yields stable, structurally defined proteins and peptides, termed Synpep 31-33. Using this approach, we constructed a Project Synthetic Proteome (PSP) derived from the intergenic regions of *Escherichia coli* K-12, generating hundreds of novel peptides with no evolutionary relationship to known proteins 34.

*E. coli* intergenic regions were selected as a concept-driven peptide reservoir rather than as an organism-specific vaccine source. These non-coding regions represent an underexplored sequence space with minimal protein-level selective constraints, enabling the generation of *de novo* synthetic peptides with a low risk of homology to host proteins. Moreover, *E. coli* provides a well-annotated and methodologically tractable reference genome for benchmarking the proposed immunoinformatics pipeline 35-39. Because mimotopes require only structural, not sequence, similarity to their target epitopes, the *PSP* dataset provides an unusually rich and unbiased search space for discovering mimics of pathogenic antigens. We therefore hypothesized that structural mimics of Aβ_42_ B-cell epitopes may exist within this synthetic peptide space.

In this study, Alzheimer's disease was used as a model system to evaluate a genome-to-mimotope discovery framework, rather than to imply any biological relationship between *E. coli* and AD pathology*.* We applied an integrated immunoinformatics and structural-bioinformatics workflow to (i) identify antigenic *Synpeps*, (ii) predict B- and T-cell epitopes, (iii) screen for three-dimensional structural similarity to Aβ_42_, and (iv) validate candidate mimotopes by docking to the Fab region of the clinically relevant 3D6 antibody (bapineuzumab). This work therefore has two objectives: first, to establish a general framework for mining non-coding DNA-derived peptides as a source of antigenic mimotopes, and second, to demonstrate its feasibility by identifying Aβ_42_-like mimotopes for Alzheimer's immunotherapy. By integrating synthetic proteomics with immunoinformatics and structural modeling, this study introduces a new paradigm in epitope-based vaccine discovery.

## Materials and Methods

### Peptide prediction from intergenic sequences and 3D structure generation

The methodology adopted for predicting novel peptides from the intergenic sequences of *E. coli* K12 substrain MG1655 genome was reported in our previous publication 40. The resulting Project Synthetic Proteome (PSP) dataset comprised predicted peptide sequences (*Synpeps*) ranging from 4 to 21 amino acids in length. For downstream analyses, including antigenicity screening, epitope prediction, structural modeling, molecular docking, and molecular dynamics simulations, only peptides within the 6-20 amino acid range were retained, as this length is optimal for linear B-cell epitope representation, mimotope stability, and reliable structural and interaction analyses.

### Antigenicity Prediction and Epitope Identification

We adopted an immunoinformatics approach to explore the therapeutic potential of the 'not-coding DNA derived peptides (*Synpeps*). The antigenicity was predicted using the Vaxijen v.2.0 server 41 which identified the probable protective antigens from the PSP dataset. Potential antigenic *Synpeps* were further studied for the presence of epitopes that may elicit a B cell response. Linear B-cell epitopes were predicted from each sequence using four different tools: Bepipred v.2.0 42, BCPred 43, ABCPred 44 and Ellipro 45 with default cutoff values for scoring the putative epitopes. Discontinuous epitopes from *Synpeps* were also predicted using Ellipro from IEDB (Immune Epitope Database https://www.iedb.org/), which accepts the peptide structure file as input. The consensus high-scoring B cell epitope from each antigenic peptide was identified by comparing the position of the epitope in the sequence predicted from all four tools considered for the study. Thus, we created a scalable virtual library of high-scoring B cell epitopes from each antigenic peptide for further analysis.

The MHC (Major Histocompatibility Complex) class I and class II binding prediction, a prerequisite for an antigenic protein to elicit T cell response, was also carried out for the antigenic PSP dataset. The online servers, NetMHC v.3.2 46 and NetMHC II v.2.2 47 were employed for identifying the peptides binding to selected human leukocyte anti-gens (HLA) Class I and Class II alleles, respectively. The selected HLA alleles were chosen based on their high prevalence and broad population coverage across diverse ethnic groups, thereby enhancing the translational relevance of the predicted epitopes. Three frequent alleles namely HLA-A*0201, HLA-B*1501, and HLA-B*2705 were considered in the human MHC Class I as they are among the most frequently reported alleles worldwide and collectively provide substantial global coverage. Similarly, HLA-DRB1*0101 was included for MHC Class II predictions due to its widespread distribution and established relevance in antigen presentation studies, ensuring meaningful representation of helper T-cell responses.

Peptides binding to the MHC II allele were sorted based on IC50 values (Table S2a-d). The binding profile of these high-confidence cross-allelic *Synpeps*, including peptides predicted to bind at least two HLA alleles across MHC class I and II, is summarized in Figure S2. In the final screening step, promising peptides that may elicit B cell response and those strongly bind the selected MHC I and II alleles or a maximum of 3 alleles with the least IC50 values were further selected as potential candidates for epitope-based vaccine design. The epitopes and IC50 values were tabulated for further study.

### Identification of mimotopes applicable in Amyloid-beta immunotherapy for Alzheimer's disease

**Figure 1:**
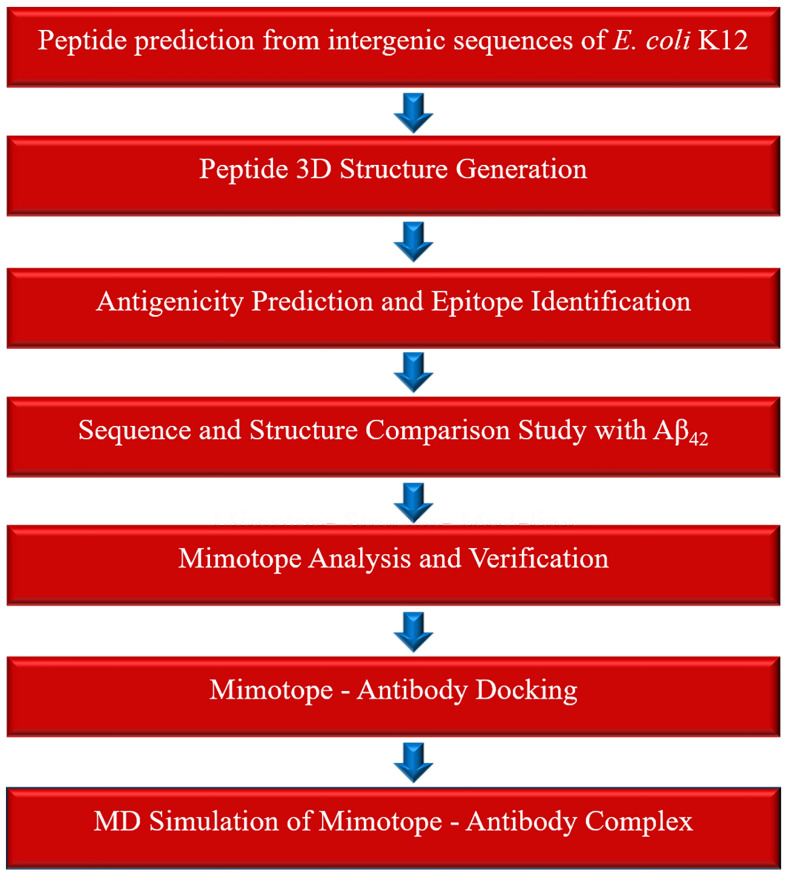
Overview of the computational workflow used to identify mimotopes applicable to amyloid-β immunotherapy for Alzheimer's disease.

We conducted an *in silico* case study to illustrate the possible applications of the epitopes predicted from the peptides of intergenic origin. Alzheimer's disease was selected as a model system for this study, as AD was reported as one of the "four big killers" that threaten the health of the elderly. Recent studies have shown that the epitopes from the toxic Amyloid beta peptide_42_ are useful as vaccines against AD 48,49, and structurally similar epitopes (mimotopes) of amyloid beta peptide_42_ can be promising in Amyloid-beta immunotherapy. We hypothesized that structurally similar epitopes of Amyloid beta epitopes may exist in our PSP dataset that can serve as potential candidate mimotopes for Alzheimer's immunotherapy. The overall workflow adopted for this study is illustrated in Figure 1.

#### Sequence and Structure Comparison Study with Aβ₄₂

A pairwise sequence and structure comparison study with Aβ₄₂ was carried out to identify the lead *Synpeps* from our PSP dataset. The sequence of toxic Aβ₄₂ was retrieved (UniportID: P05067) and sequence-based pairwise comparison was done with our PSP dataset using BLASTP 50. The solution structure of Aβ₄₂ was downloaded from the PDB database (PDB ID: 1IYT) and compared with each of the 3D structures of *Synpeps*. Pairwise-Dali server 51 and FATCAT server 52 were employed for the structural comparison studies. To account for the limited conformational space of short peptides, we applied strict filters to reduce false positives. Only structural matches identified by both FATCAT (P < 0.01) and DALI (Z-score ≥ 2.0) with RMSD < 3.0 Å were considered. Additionally, aligned regions were required to overlap with predicted B-cell epitopes, particularly in the immunodominant N-terminal region of Aβ₄₂, to enhance biological relevance. As mimotopes must have structural similarity and potential B cell epitopes, *Synpeps* with structural similarity to Aβ₄₂ were selected and their epitopes were identified. The predicted epitopes derived from the Aβ₄₂ peptide were used for comparative analysis, and the precise regions of sequence identity between Aβ₄₂ and our *Synpeps* were accurately verified. The best-matching mimotopes were identified and tabulated for further study.

#### Mimotope Analysis and Verification

The identified mimotopes were further analyzed for their novelty and uniqueness. A sequence search by BLASTP (with parameters specific to short peptide sequences, scoring matrix-PAM30; E-value 20000 and word size 2) was performed to test any significant matches against human proteins from the non-redundant sequence. Epitope similarity search against the Immune epitope database (IEDB) and PepBank database (http://pepbank.mgh.harvard.edu/) was also done. The 3D structural homology mapping tool from IEDB was also used to map the source epitope sequence to a similar PDB structure. This step helps to identify any similar proteins in PDB. The essential physicochemical parameters of the mimotopes were also predicted and compared with those of Aβ₄₂.

#### Mimotope Structure Modeling and Mimotope -Antibody Docking

The mimotopes were modeled using PEP-FOLD v.3.0 53 and I-TASSER v.5.2 servers 54. All modeled peptide structures were subjected to energy minimization using default parameters in the I-TASSER and PEP-FOLD pipelines prior to structural comparison and molecular docking, to ensure conformational stability and reliable interaction analysis. Molecular docking studies were then carried out to analyze the ability of the mimotopes to bind Fab (Fragment antigen-binding) of a monoclonal antibody, thereby identifying the most interacting mimotope-Fab complex. The structure of Fab (Fragment anti-gen-binding) of 3D6 complexed with an antigenic amyloid epitope 55 was downloaded from PDB (PDB id: 4ONF) as the target structure, and our mimotopes were used as ligands for docking studies. 4ONF is a reported crystal structure of a recombinant Fab fragment of 3D6 in complex with Aβ1-7 solved at 2.0 Å resolution. This report confirms structural conservation between the parent murine antibody and its humanized version - Bapineuzumab (humanized 3D6), a neo-epitope-specific antibody recognizing Aβ1-5. The ZDOCK program v.3.0.2 56 integrated with the Biovia Discovery Studio v.4.0 suite 57 and PatchDock server 58 were employed to study the molecular interaction between our mimotopes and the 4ONF. The mimotopes were superimposed on the native ligand (Aβ₄₂ epitope) of the crystal structure to confirm the structural similarity, and the RMSD was noted. The native ligand was re-docked to the receptor structure to confirm the binding and to analyze the binding site residues. The binding site occupied by the native ligand was further considered to dock our mimotopes. The docked poses from ZDOCK were reranked using the energy function of ZRANK program 59, whereas the initially docked complexes from PatchDock were refined and reranked using the Fire-Dock program 60 based on the global energy (binding energy). The near-native con formations of the docked complexes were visualized using Biovia discovery studio v.4.0 visualizer and were further closely analyzed for favorable intermolecular interactions. The type of interactions such as hydrogen bonds, their corresponding bond lengths, non-bond interactions, and the interacting residues were noted and tabulated. Based on the docking scores, energy values, and molecular interactions, the best mimotope- Fab complex was identified and studied for its stability using molecular dynamic simulation.

#### Molecular Dynamic Simulation of Mimotope -Antibody Complex

The best-scoring docked complex was selected for molecular dynamics simulations using the GROMACS 2021 package, executed in CPU-only mode without GPU acceleration. All simulations were performed on a Windows-based workstation employing a multi-core CPU environment for the production run 61 with CHARMm 27 force field. The complex was placed at the center of a rhombic dodecahedral box, and the distance to the edge of the box was set to 1.5 nm. The box was solvated using TIP3 water molecules. After solvation, the system was neutralized by adding an appropriate number of counter ions, sodium (Na+) and chloride (Cl-). An energy minimization step was performed in the solvated neutralized system to eliminate the inappropriate geometry. The energy minimization was performed using the steepest-descent algorithm of 50,000 steps for each system. After minimization, the first equilibration of the system was performed with a constant number of particles and volume, and the second equilibration with temperature (NVT) and a constant number of particles, pressure, and temperature (NPT). The standard temperature of the system was maintained at 300 K by applying a Berendsen thermostat, and the system's pressure was maintained using the Parrinello-Rahman coupling method. After the equilibration step of NVT and NPT, the production MD run was performed for 100 ns. The trajectory coordinates were written for every 2 fs to get the maximum number of frames for analysis. After the simulation, the trajectory analysis was performed to understand the stability of the complex. The Root Mean Square Deviation (RMSD) plots were generated using the software QTGrace (https://sourceforge.net/projects/qtgrace/). The residue-wise root mean-square fluctuation (RMSF), Radius of gyration and hydrogen bonds analysis were also performed. Molecular docking and molecular dynamics simulations were executed in CPU-only mode using GROMACS for 100 ns runs.

## Results

### *Synpeps* generated from intergenic regions

The computational translation of intergenic regions of *E. coli* K12 substrain MG1655 genome generated 424 novel peptides named '*Synpeps*', which were further considered for antigenicity prediction.

### Antigenic epitopes predicted from *Synpeps*

The Vaxijen server predicted around 50.7% (215 peptides) of our *Synpeps* to be probable antigens based on Vaxijen scores. The distribution of VaxiJen antigenicity scores across the complete *Synpep* dataset, highlighting the antigenicity threshold (0.5) used for downstream filtering, is shown in Figure S1. The linear B cell epitopes predicted by the four different tools BepiPred, BCPred, ABCpred, and Ellipro, were compared based on the confidence scores and the position in the sequence. Out of 424 *Synpeps*, BepiPred predicted 58%, BCPred -70%, ABCPred - 98%, and Ellipro predicted 95% of proteins to possess linear B cell epitopes. A total of 415 *Synpeps* were predicted to have discontinuous epitopes in the Ellipro tool. The high-scoring consensus epitopes from each antigenic protein were selected and tabulated. Table S1 (Supplementary material) shows the high-scoring consensus B cell epitopes and their predicted confidence scores for our 215 antigenic *Synpeps*.

The binding affinity of *Synpeps* towards the 3 most common human MHC I (Human Leukocyte Antigens-HLA) alleles, namely HLA-A*0201, HLA-B*1501, and HLA-B*2705, was predicted and tabulated based on IC50 values. Strong binders of MHC II allele HLA-DRB1*0101 with IC50 values less than 50nM were also sorted out. 42 *Synpeps* out of our PSP dataset were found to be promising leads that may elicit a T cell response. Three *Synpeps* (PSP35, PSP297, and PSP349) were found to bind all 4 alleles strongly, whereas 39 *Synpeps* were found to bind at least 3 alleles strongly. The remaining pep-tides were found to be weak or non-binders. Table S2(a) to S2(d) (Supplementary material) shows the epitopes of lead *Synpeps* with the MHC I and MHC II alleles they strongly bind.

### Mimotope identification for AD immunotherapy

Our *in silico* case study to identify the potential mimotopes of amyloid beta_42_ peptide, from the *PSP* dataset offered 7 promising lead peptide candidates for Alzheimer's immunotherapy.

#### Sequence and Structure Comparison Study with Aβ₄₂

The pairwise sequence similarity analysis between the toxic amyloid-β₄₂ (Aβ₄₂) peptide and the *Synpeps* identified eight sequence-level matches. However, both the percentage identity and alignment lengths were statistically insignificant (Table S3, Supplementary material). In contrast, the 3D structural comparison yielded more promising results , 115 significant structural matches were detected using FATCAT (P < 0.05), and 19 significant hits were identified with the DALI server based on Z-scores, suggesting notable structural resemblance between several *Synpeps* and Aβ₄₂. Structures of homologous proteins typically get higher similarity scores in Dali predictions than the structures of evolutionarily unrelated proteins. Structures with Z scores of 2 or more were significantly similar and had similar folds. The 19 structural mimics predicted by both servers were further considered for the study. The predicted P value, Z score, RMSD, and structurally equivalent positions were tabulated (Table 1).

Of the 19 structural mimics, 10 were potential antigens based on our Vaxijen prediction results. To further propose the identified 10 *Synpeps* as potential functional mimics of Aβ₄₂ peptide and source of mimotopes, the presence of B cell epitope is also essential in the structurally aligned regions of the peptide. The peptide B cell epitope prediction results were closely analyzed and compared to those of Aβ₄₂. The N-terminal region of Aβ₄₂ (Aβ₁-₁₅) constitutes an immunodominant and conformationally accessible B-cell epitope that is preferentially targeted by therapeutic antibodies due to its surface exposure on synaptotoxic Aβ oligomers and protofibrils. Its validated role in antibody-mediated Aβ clearance is underscored by its incorporation into second-generation peptide vaccines (e.g., UB-311, ACI-24, CAD106), which elicit oligomer-neutralizing antibodies while minimizing T-cell autoreactivity 62, 63, 64, 65. Subsequently, we predicted the B cell epitope of Aβ₄₂ using the same set of tools selected for this study, and the results were consistent with the published data (Table S4 - Supplementary material). Of our 10 *Synpeps*, seven were found to have B cell epitopes in the structurally aligned N-terminal regions of Aβ₄₂, whereas the other 3 had the B cell epitope in the unaligned C terminal ends. Therefore, the B cell epitopes of the 7 *Synpeps* were further selected as the best mimotopes for AD immunotherapy.

#### Mimotope Analysis and Verification

The physicochemical properties of the selected mimotopes, including molecular weight (Da) and isoelectric point (pI), were computationally predicted. The sequence-specific BLASTP search revealed no significant similarity to any known human proteins, as all retrieved matches showed high E-values and were deemed non-significant (Table S5, Supplementary Material). Furthermore, epitope similarity searches against the Immune Epitope Database (IEDB) and PepBank returned no hits. Similarly, the IEDB homology mapping tool, used to identify structural homologs in the Protein Data Bank (PDB), yielded no significant matches. Collectively, these findings suggest that the selected mimotopes are unique sequences with no detectable similarity to previously reported human or PDB proteins.

#### Molecular structure modeling and docking between mimotopes and Fab

**Figure 2:**
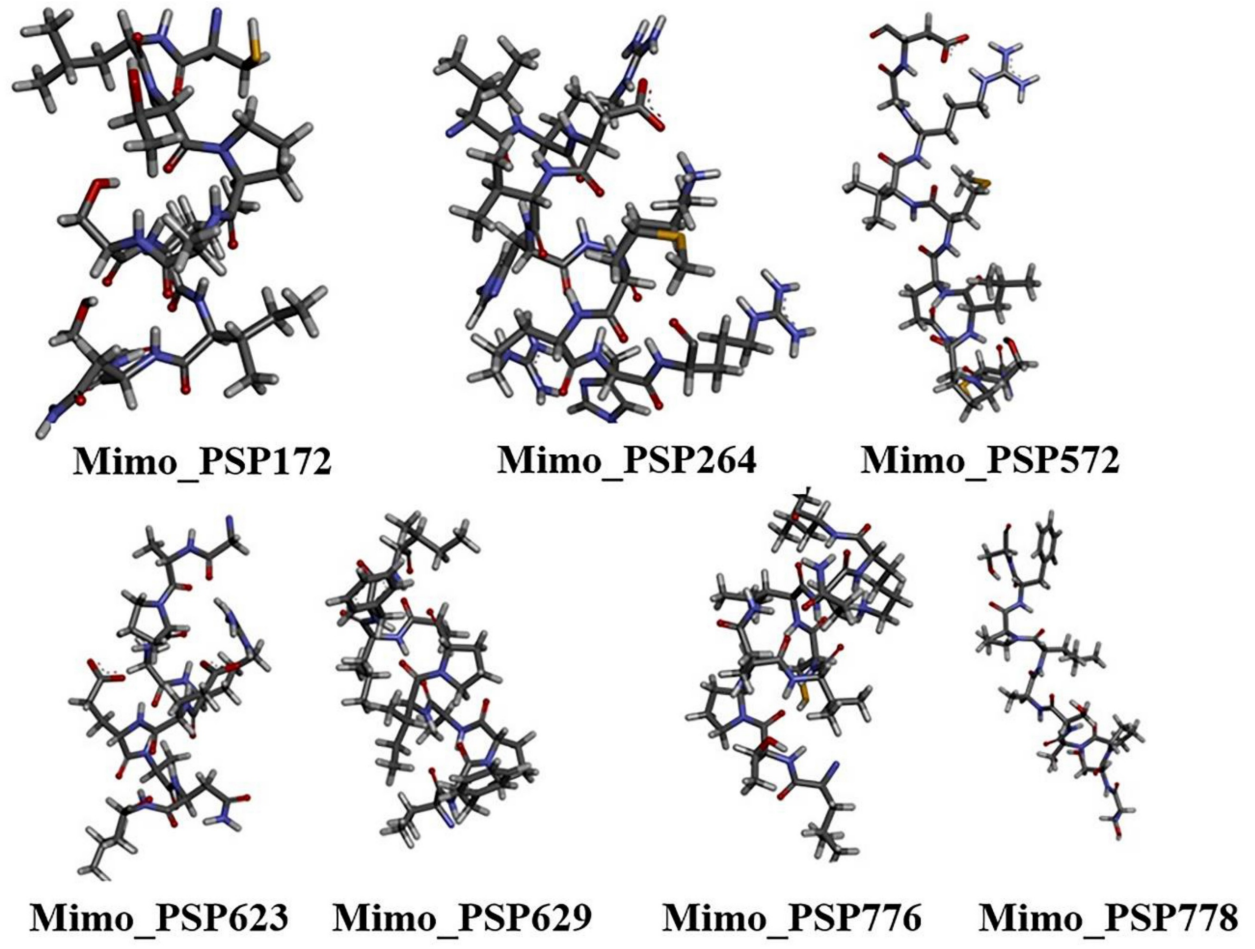
Ab initio modeled three-dimensional structures of selected mimotopes derived from *Synpeps*. Structures were generated using I-TASSER and PEP-FOLD and are shown in stick representation with CPK coloring, illustrating the conformational diversity of candidate mimotopes prior to docking analysis.

The top 3D structural models of the seven mimotopes (Figure 2), generated *ab initio* using the I-TASSER and PEP-FOLD servers, were selected for subsequent molecular docking analyses.

The modeled mimotopes (10aa) were found to have significantly low RMSD values when superimposed to the Aβ 1-7 region (native ligand) of the target 3D structure (4ONF). The RMSD for superimposition and the I-TASSER C scores for mimotope modeling were tabulated (Table S6 - Supplementary Material). The crystal structure of Aβ_1-7_ epitope bound with Fab of 3D6 (4ONF) when analyzed using Biovia Discovery Studio 4.0 showed 28 favorable non-bond interactions. Three salt bridges were seen along with several hydrogen bonds. The residues Asp31 and Asp33 in the light chain of Fab are involved in salt bridge formation with Arg5 of the epitope and Arg101 formed a salt bridge with Glu3 of the epitope. The native ligand, when redocked to Fab, most of the interactions involving the residues Asp1, Glu3, Phe4, and Arg 5 were found to be retained. Two salt bridges were found to be retained between Asp31 with Arg5 and Arg101 with Glu3, whereas an electrostatic interaction was shown between Asp33 and Arg5.

Among the mimotopes, several candidates demonstrated binding affinities comparable to or exceeding that of the native epitope (Table 2). Notably, Mimo_PSP572 displayed the most favorable ZRANK score (-92.01), surpassing the native ligand, indicating a highly stable interaction profile. Mimo_PSP629 also showed a ZRANK score (-86.39) comparable to the native Aβ epitope, while maintaining a favorable FireDock global energy (-56.10). These results suggest that both PSP572 and PSP629 form energetically stable complexes within the Fab binding pocket. FireDock global energy analysis further revealed that several mimotopes exhibited more favorable binding energies than the native ligand. For instance, Mimo_PSP778 showed the lowest global energy (-58.81), followed by *Mimo_PSP172* (-58.70) and *Mimo_PSP623* (-57.58), indicating strong predicted binding despite moderate ZDOCK scores. This highlights that favorable binding energy can arise from optimized electrostatic and hydrogen-bond networks rather than shape complementarity alone. Interaction counts provide additional insight into binding quality. *Mimo_PSP264* formed the highest number of favorable interactions (30 interactions), substantially exceeding the native epitope, and established a critical salt bridge between Arg8 (PSP264) and Asp31 (Fab)—a residue central to native Aβ recognition. Similarly, Mimo_PSP572 and *Mimo_PSP623* formed salt bridges with Asp31 and Arg101, respectively, mirroring key contacts observed in the native Aβ₁-₇-Fab complex (Table S7). These conserved interactions strongly support native-like antibody engagement.

Overall, the docking results indicate that the selected mimotopes occupy the same antigen-binding pocket as the native Aβ₁-₇ epitope and establish comparable or enhanced interaction networks with the Fab fragment. While individual mimotopes vary in ZDOCK, ZRANK, and interaction counts, their combined docking metrics demonstrate energetically stable, structurally compatible, and interaction-rich binding modes, consistent with functional epitope mimicry.

#### Molecular dynamic simulation of mimotope-Fab complex

**Figure 4:**
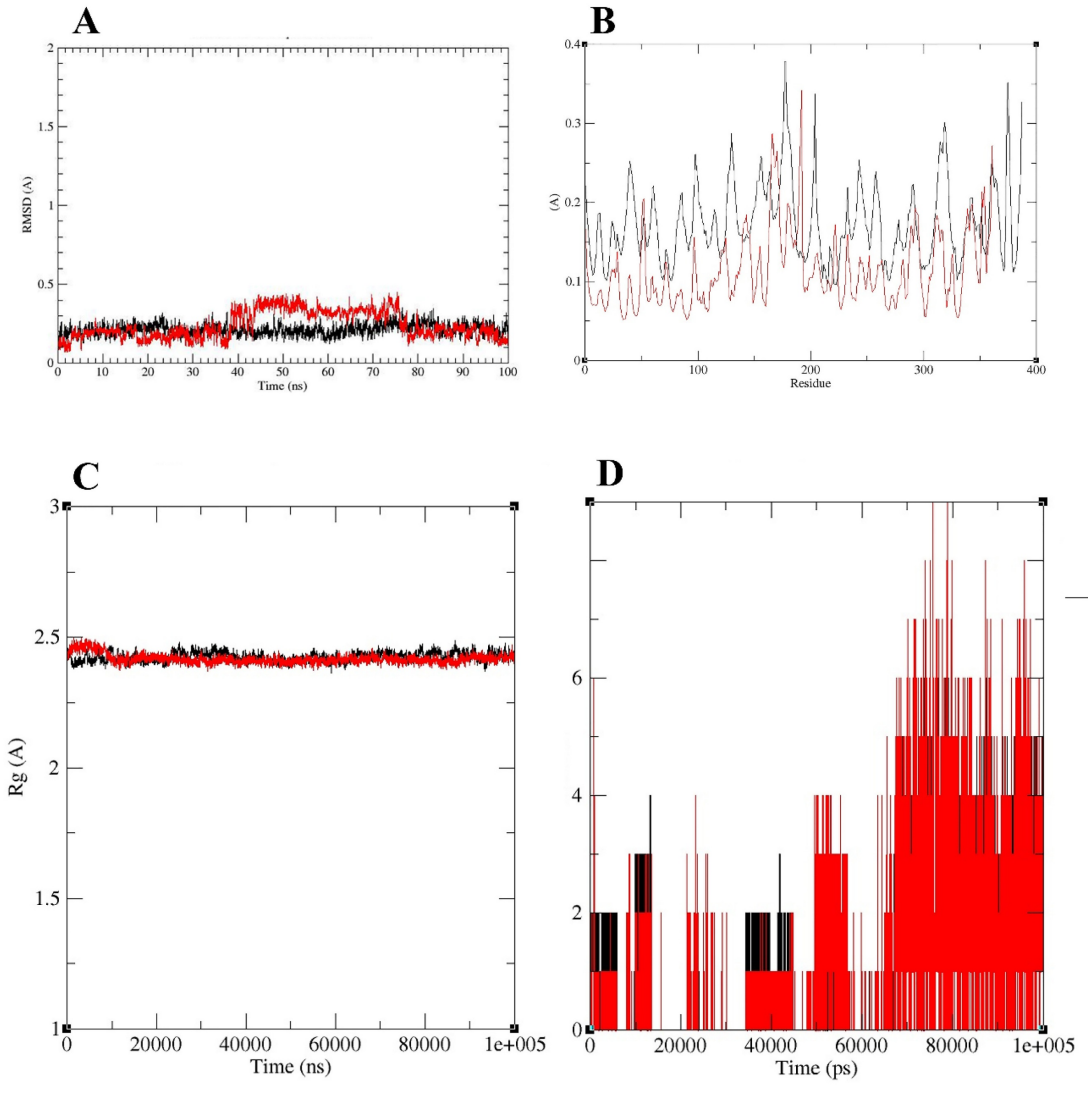
Molecular dynamics (MD) analysis of the mimo_PSP572-Fab (3D6; PDB ID: 4ONF) complex. (A) RMSD of the Fab domain (black) and mimo_PSP572 peptide (red) during the simulation. (B) RMSF comparison of the mimo_PSP572-Fab complex and the Fab domain alone. (C) Radius of gyration (Rg, Å) of the Fab (black) and mimo_PSP572-Fab complex (red). (D) Time evolution of intermolecular hydrogen bonds over the 100 ns MD simulation, showing the number of hydrogen bonds (black) and interacting pairs within 0.35 nm (red).

Molecular dynamics simulation studies confirmed the stability of the best-docked complex (with the highest ZRANK score). The RMSD analysis was conducted to understand the stability and flexibility of the mimo_PSP572 -Fab complex. The RMSD plot of the target protein (Fab domain of 3D6 in the apo form (4ONF)) showed that the plot attained a steady stage at 100 ns without much fluctuation (Figure 4A). The complex attained equilibration till 40 ns, a slight fluctuation was observed from 40 ns, and the steady state was attained from 80 ns. The residue fluctuation was between 0.1 Å and 0.4 Å reflecting a transient conformational adjustment before re-stabilizing. From the RMSD analysis, it was evident that the mimotope-Fab complex was highly stable with no evidence of major structural disruption during the simulation.

The residue-wise root mean square fluctuation (RMSF) analysis was performed to evaluate the structural flexibility of the Fab domain of 3D6 in the apo form (4ONF) and in complex with *Mimo_PSP572* (Figure 4B). Overall, the apo Fab exhibited higher fluctuations across most residues compared to the ligand-bound complex, indicating increased structural flexibility in the absence of the ligand. In the apo structure, several regions showed pronounced fluctuations, with RMSF values reaching up to ~0.35-0.38 Å, particularly around residues 150-200 and 330-380 (Figure 4B). These peaks suggest the presence of flexible loop regions that may contribute to conformational instability when the Fab is unbound. In contrast, the *Mimo-PSP572*-bound complex displayed consistently lower RMSF values throughout the protein, mostly remaining below ~0.20 Å (Figure 4B). Notably, residues corresponding to highly flexible regions in the apo form exhibited reduced fluctuations upon ligand binding, indicating ligand-induced stabilization. The suppression of large-amplitude motions in these regions suggests stronger structural rigidity and enhanced conformational stability of the Fab upon interaction with *Mimo-PSP572*. Overall, the RMSF results demonstrate that binding of *Mimo-PSP572* significantly reduces residue-level fluctuations of the Fab domain, supporting the stabilizing effect of the ligand on the protein structure during the simulation.

The Radius of Gyration (Rg) analysis showed that both systems maintained highly stable Rg values centered around ~2.4-2.5 Å throughout the trajectory, indicating preservation of overall compactness and absence of large-scale unfolding or structural expansion (Figure 4C). Minor early fluctuations were observed for *Mimo-PSP572*, followed by convergence with the Fab of 3D6, after which both profiles closely overlap with minimal variance. The analysis suggested that the two systems exhibit comparable global structural stability and compactness over the course of the simulation.

Hydrogen bond analysis was carried out to evaluate the stability and persistence of interactions between the target protein and ligand throughout the molecular dynamics simulation (Figure 4D). The number of hydrogen bonds and interacting pairs within a cutoff distance of 0.35 nm were monitored over the entire simulation time of 100 ns. During the initial phase of the simulation (0-20 ns), the complex exhibited a low and fluctuating number of hydrogen bonds, typically ranging between 0 and 2 (Figure 4D). This behavior indicates an initial equilibration phase, during which the ligand undergoes conformational adjustment within the binding pocket. Between 20 and 60 ns, a gradual increase in hydrogen bond formation was observed, with intermittent fluctuations reaching up to 3-4 hydrogen bonds. This period suggests progressive stabilization of the protein-ligand complex as favorable interactions begin to form. Notably, after approximately 60 ns, the number of hydrogen bonds increased significantly and remained relatively stable for the remainder of the simulation. During this phase, the complex consistently maintained 3-6 hydrogen bonds, with occasional peaks reaching up to 8-9 interactions. The sustained presence of multiple hydrogen bonds indicates strong and stable protein-ligand interactions. Additionally, the number of interacting pairs within 0.35 nm closely followed the hydrogen bond trend, further confirming tight binding and close contact between the target protein and ligand.

## Discussion

Given the global burden of Alzheimer's disease (AD) and the limited therapeutic options, we selected AD as a model system for a case study. Recent reviews emphasize that mimotopes of toxic amyloid-β (Aβ₄₂) peptides represent a promising strategy for safe epitope-based vaccines, capable of preventing Aβ accumulation even in healthy individuals. Guided by this rationale, we searched for structural mimics of Aβ₄₂ within our *Synpeps* dataset. Similar approaches have successfully identified therapeutic mimotopes for autoimmune diseases 66. The observation that *Synpeps* may resemble known antigenic peptides structurally or functionally provides novel insights into their potential roles and applications in immunotherapy. Active immunization against amyloid-β (Aβ) remains a major investigational strategy for Alzheimer's disease, with several peptide-based vaccines advancing through clinical development. Second-generation vaccines are designed to elicit B-cell responses to immunodominant regions of Aβ while minimizing harmful T-cell activation and inflammation that plagued early candidates such as AN-1792. For example, UB-311 comprises two synthetic Aβ1-14 B-cell epitopes each linked to distinct helper T-cell peptide sequences (UBITh®), and has demonstrated robust anti-Aβ antibody generation with high responder rates and favorable safety in Phase I/II trials 63. CAD106 uses a short Aβ1-6 peptide conjugated to a bacteriophage Qβ virus-like particle to improve immunogenicity while avoiding Aβ-specific T-cell activation, showing acceptable safety and serological responses in phase II clinical studies 64. ACI-24 represents another approach using Aβ1-15 peptides anchored in liposomes to induce β-sheet-targeted antibodies, with ongoing early-phase trials exploring safety, tolerability, and anti-Aβ immunogenicity 65. These established peptide vaccines share a classical design paradigm: they are derived directly from known antigenic fragments of the target protein (Aβ) and often include exogenous T-cell help or particulate carriers to enhance inherent immunogenicity. Mechanistically, this strategy relies on sequence-based epitope recognition and immune presentation of known pathogenic regions of Aβ to stimulate adaptive antibody responses. In contrast, our non-coding DNA mimotope strategy described here does not start with known pathological antigen fragments. Instead, it systematically mines synthetic peptides derived from non-coding genomic sequence space (e.g., intergenic regions), identifying peptides that structurally mimic conformational features of relevant B-cell epitopes through computational screening, rather than relying on direct sequence homology. Because mimotopes are selected for structural and functional resemblance to target epitopes, they can theoretically engage the same paratope footprints as native sequences without sharing primary sequence identity, potentially reducing autoimmune risk and expanding the repertoire of candidate vaccine immunogens beyond naturally occurring peptide fragments. This strategy is mechanistically distinct from both classical peptide vaccines and recombinant constructs, as it leverages *de novo* structural mimicry rather than native antigen sequence mimicry.

Moreover, traditional peptide vaccines must balance immunogenicity, T-cell help, and safety through empirical design of carriers, adjuvants, and epitope selection. Despite improvements, second-generation Aβ vaccines have not yet demonstrated robust clinical efficacy, and their antibody responses can wane over months after the last injection. The non-coding DNA mimotope paradigm offers a potentially broader structural space for mimicking not only linear sequences but also conformational surfaces associated with pathological species such as oligomers — a property that may be underutilized in current vaccine designs.

Recent advances have demonstrated that many sequences previously classified as non-coding actually encode functional peptides. For instance, short bioactive peptides derived from short open reading frames (sORFs) in *Drosophila melanogaster* regulate transcriptional activity, despite being initially annotated as non-coding RNAs 67. The development of new tools to identify such sORFs highlights the rapid progress in this field 68. In our study, we focused on ORFs coding for proteins longer than 100 amino acids, although numerous sORFs remain under investigation. Some putative ORFs identified from our dataset possess coding potential but were overlooked during genome annotation. By integrating immunoinformatics and structural bioinformatics**,** our approach could identify mimics of neo-epitopes that are poorly represented in natural peptide libraries or truncated sequences.

Importantly, because mimotopes are defined by structural and epitope mimicry rather than evolutionary homology, there is no requirement that their genomic origin be biologically related to the target antigen. Alternative sequence sources, such as the human genome, pseudogenes, or the microbiome, would introduce substantial risks of immunological cross-reactivity, immune tolerance, or autoimmunity, whereas bacterial non-coding DNA offers a largely orthogonal sequence space that minimizes homology to human proteins while maximizing diversity. Accordingly, the present study should be interpreted as a conceptual and methodological demonstration of a genome-to-mimotope pipeline rather than as a direct translational vaccine product; once validated, the same framework could be applied to any genomic source optimized for safety, manufacturability, and clinical use.

Although the present work is computational, immunogenicity and safety considerations are central to the translational feasibility of mimotope-based vaccines. A primary concern is immune cross-reactivity with endogenous human proteins, which could lead to tolerance or autoimmunity. To address this, all selected mimotopes were screened against the human proteome using BLASTP with parameters optimized for short peptides, and no statistically significant matches were detected, indicating minimal risk of molecular mimicry with self-antigens. In addition, epitope similarity searches against the Immune Epitope Database (IEDB) and PepBank returned no matches, and structural homology mapping against the Protein Data Bank revealed no known immune epitopes, collectively suggesting that these peptides occupy a novel antigenic space. An additional consideration relates to the intrinsic structural polymorphism of Aβ₄₂, which is known to adopt monomeric, oligomeric, and fibrillar conformations. In this study, the monomeric NMR structure of Aβ₄₂ (PDB ID: 1IYT) was used as a reference scaffold because the analysis focused on the immunodominant N-terminal region (Aβ₁-₁₅), which remains solvent-exposed and antibody-accessible across diverse Aβ assemblies. Although aggregation-dependent conformational variability may influence epitope presentation, the present approach prioritizes broad epitope mimicry rather than conformation-specific binding; future extensions of this framework may incorporate oligomeric and fibrillar Aβ models to assess conformation-dependent mimotope specificity. We also acknowledge that the antigenicity and B-cell epitope predictions in this study rely on computational tools such as VaxiJen and sequence-based epitope predictors, which have limited accuracy for short peptides. Accordingly, the proposed mimotopes should be regarded as hypothesis-generating candidates. Moreover, structural similarity to Aβ₄₂, while supportive of potential epitope mimicry, does not by itself establish functional equivalence, and their immunogenic potential and ability to elicit Aβ₄₂-specific antibody responses must be confirmed experimentally. Our antigenicity predictions revealed that approximately 50% of peptides from non-coding regions are probable antigens. If expressed in bacteria, these proteins could function as protective antigens. Currently, the Protegen database lists only 17 protective antigens from the E. coli genome; our predicted antigens, upon validation, could expand this repertoire. Importantly, because the mimotopes are derived from bacterial non-coding DNA rather than expressed bacterial proteins, they are not subject to immune tolerance arising from natural host-microbe exposure. Instead, they behave as synthetic neo-epitopes whose immunogenicity is driven by structural mimicry of Aβ rather than sequence homology to either host or microbial antigens. Nevertheless, like all peptide vaccines, these candidates will require empirical validation for autoreactivity, cytokine skewing, and off-target binding *in vitro* and *in vivo* before clinical translation.

Another potential application of our epitope library is in epitope-tagging techniques 69, 70, where known epitopes are fused to recombinant proteins to enable detection when antibodies are unavailable. Evaluating immunogenicity and safety is crucial for therapeutic development 71. In this study, our in- silico T-cell epitope predictions provide a preliminary assessment of the immunogenic potential of peptides derived from intergenic regions. Peptide-MHC binding affinity serves as a key indicator for vaccine design. Furthermore, non-immunogenic peptides can be repurposed for structure-based drug design. Collectively, our virtual library of synthetic peptides offers a resource for screening candidate molecules with diverse therapeutic applications.

To our knowledge, this is the first study to propose intergenic regions as a source of potential therapeutic mimotopes. Our findings highlight that structural similarity can exist in the absence of sequence similarity, as demonstrated by the conformational resemblance between Aβ₄₂ and *Synpeps*. While these mimotopes will require high-quality adjuvants and carriers to function effectively as vaccines, future *in vitro* and *in vivo* validation is essential. Comparative studies assessing the immunogenicity of promising *Synpeps* versus known therapeutic peptides, as well as exploring non-antigenic *Synpeps* as potential drug candidates through virtual screening, represent promising directions for further research.

## Conclusions

Using Alzheimer's disease as a model, this study identified seven promising mimotope candidates derived from non-coding DNA sequences that exhibit notable structural and functional resemblance to the pathogenic amyloid-β₄₂ (Aβ₄₂) peptide. Molecular docking with the Fab region of the 3D6 antibody revealed binding affinities comparable to the native Aβ₁-₇ epitope, while molecular dynamics simulations confirmed the stability of the resulting peptide-antibody complexes. These findings highlight the potential of repurposing non-coding genomic regions as a previously untapped source of therapeutic peptides. Further *in vitro* and *in vivo* validation is essential to establish the immunogenicity and efficacy of these mimotopes. In conclusion, this work presents a novel immunoinformatics-driven framework for converting non-coding DNA into synthetic peptide leads with potential applications in epitope-based vaccine design and immunotherapy.

## Supplementary Material

Supplementary figures and tables.

## Figures and Tables

**Figure 3:**
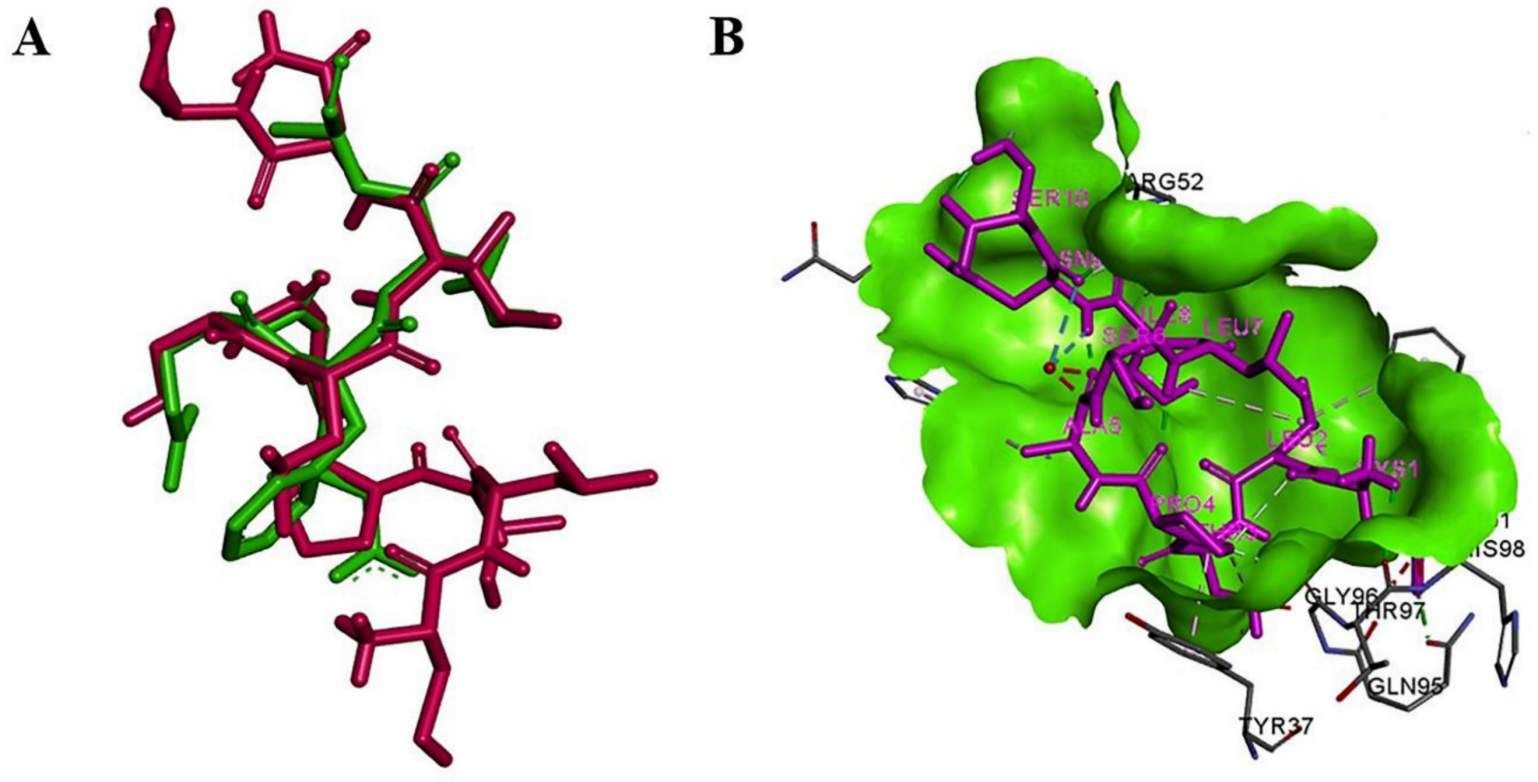
Structural comparison and interaction of mimo_PSP572 with Aβ₄₂ epitope and antibody Fab region (A) Superimposition of the mimotope (magenta) and native Aβ₄₂ epitope (green) showing structural overlap (RMSD = 0.44), suggesting conformational mimicry (B) Binding pose of mimo_PSP572 (magenta) within the Fab binding site (green surface) of the 3D6 antibody (PDB ID: 4ONF), highlighting key interacting residues and favorable binding interactions.

**Table 1:** Structural comparison between *Synpeps* and Aβ₄₂.

*Synpep* ID	P value (Fatcat)	Z- score (Dali)	RMSD (Fatcat/Dali)	Equivalent positions (Fatcat/Dali)		
PSP112	0.00142	2.4	1.74/6.0	40/38		
PSP172	0.00221	2.3	1.96/5.4	41/37		
PSP233	0.00225	2.2	2.87/4.2	33/35		
PSP264	0.00326	2.1	2.89/4.3	32/34		
PSP328	0.00285	2.5	1.68/5.0	35/38		
PSP446	0.000536	2.4	1.14/2.6	23/35		
PSP450	0.00185	2.1	1.98/5.0	29/35		
PSP461	0.00145	2.1	1.93/4.1	30/35		
PSP501	0.00114	2.2	1.72/3.6	29/33		
PSP542	0.00755	2.2	3.21/3.9	31/33		
PSP572	0.00545	2	1.22/3.5	30/33		
PSP623	0.00659	2	2.63/3.3	27/33		
PSP629	0.00379	2.1	2.51/3.8	30/32		
PSP669	0.00419	2	2.43/4.0	29/32		
PSP702	0.000458	2.1	1.32/4.1	24/32		
PSP734	0.00158	2.2	1.79/3.0	25/31		
PSP769	0.00444	2.1	2.48/3.3	29/31		
PSP776	0.00244	2.2	2.18/3.0	30/30		
PSP778	0.00259	2.3	2.48/2.4		31/31	
						

**Table 2:** Docking scores for mimotopes against Fab of 3D6 (4ONF).

Ligands	ZDOCK score	ZRANK score	Global energy (FireDock)	No. of favorable interactions
Aβ epitope	11.52	-86.231	-53.72	18 (Salt Bridge: Arg5: L:Asp31)
Mimo_PSP172	9.16	-78.299	-58.7	19
Mimo_PSP264	8.44	-49.119	-35.12	30 (Salt Bridge: Arg8:L: Asp31)
Mimo_PSP572	7.42	-92.014	-43.11	26 (Salt Bridge: Cys1:L: Asp31)
Mimo_PSP623	9.8	-71.723	-57.58	20 (Salt Bridge: Arg5 :L: Asp31)
Mimo_PSP629	9.84	-86.392	-56.10	14
Mimo_PSP776	10.14	-64.491	-51.60	16
Mimo_PSP778	8.36	-79.553	-58.81	12

## Data Availability

The data presented in this study are available on request from the corresponding author.
