# Supplementary material for: Non-Coding DNA-Derived Mimotopes of Aβ₄₂ as Novel Candidates for Alzheimer's Peptide Vaccine Design
Source: Int J Med Sci. 2026 Mar 4;23(4):1320–32. doi: 10.7150/ijms.127358 (PMC13048884; doi:10.7150/ijms.127358)
Supplement: Supplementary file 1 — Supplementary figures and tables. [file ijmsv23p1320s1.pdf]

# Supplementary Data

Table S1: Vaxijen scores and B cell epitope prediction scores for 215 predicted antigenic *Synpeps*

| PSP ID | *Synpep* Epitope Sequence | Start position | End position | Number of Residues | Vaxijen score | Predicted high scores | | | |
|---|---|---|---|---|---|---|---|---|---|
| | | | | | | Bepi Pred | BC Pred | ABC Pred | Ellip ro |
| 1 | MPAVSFCCKTIHAA | 16 | 29 | 14 | 0.6652 | NA | **0.83** | 0.77 | 0.818 |
| 8 | VTRNGGNCQPTRRRKSPNT NKKGTSVD | 22 | 48 | 27 | 0.698 | **1.928** | 1 | 0.8 | 0.658 |
| 11 | SGCKPYVPVMSLTA | 34 | 47 | 14 | 0.6945 | 0.617 | **0.839** | 0.74 | 0.641 |
| 26 | TQFILNSNNWLLRL | 26 | 39 | 14 | 0.6614 | NA | **0.978** | 0.74 | 0.652 |
| 30 | KEKSSYQTEDEKRGN | 15 | 29 | 15 | 0.7973 | **1.886** | 1 | 0.73 | 0.789 |
| 33 | FYVDESSSRCFAPY | 14 | 27 | 14 | 0.5698 | NA | **0.991** | 0.68 | 0.671 |
| 35 | SCTEWPTDRR | 1 | 10 | 10 | 0.5742 | **1.449** | 0.997 | 0.82 | 0.687 |
| 71 | GVSDRS | 9 | 14 | 6 | 1.0369 | **0.973** | 0.937 | 0.8 | 0.703 |
| 73 | LITNCVDSCCKETL | 37 | 50 | 14 | 0.6103 | NA | **0.913** | 0.67 | 0.682 |
| 86 | QKSSD | 32 | 36 | 5 | 0.7509 | **0.807** | 0.781 | 0.69 | 0.668 |
| 89 | CKSKPTKINSVSQGD | 5 | 19 | 15 | 0.5001 | **1.137** | 0.954 | 0.77 | 0.683 |
| 98 | HKRHPTFQRESPANTGGL | 30 | 47 | 18 | 0.5239 | **1.814** | 0.993 | 0.76 | 0.742 |
| 118 | QERTWNP | 28 | 34 | 7 | 0.5110 | **1.003** | 0.908 | 0.74 | 0.711 |
| 122 | TRFSPQIEQGYHVK | 17 | 30 | 14 | 0.5869 | 0.797 | **0.98** | 0.73 | 0.806 |
| 130 | INVNSAAGRAPTAI | 30 | 43 | 14 | 0.7043 | **1.359** | 0.995 | 0.84 | 0.624 |
| 142 | EINHFACHSTTGTL | 31 | 44 | 14 | 0.6232 | NA | **0.998** | 0.74 | 0.742 |
| 148 | KPGKSGPRRSL | 13 | 23 | 11 | 0.8525 | **2.196** | 0.918 | 0.76 | 0.645 |
| 150 | YFCLHFIQIL | 12 | 21 | 10 | 0.6335 | NA | 0.769 | **0.85** | 0.736 |
| 155 | LRNSA | 1 | 5 | 5 | 0.5470 | 0.651 | NA | 0.66 | **0.677** |
| 164 | RNIRPQGNDNG | 33 | 43 | 11 | 0.5573 | **2.391** | 0.889 | 0.82 | 0.767 |
| 170 | NGGTDS | 21 | 26 | 6 | 1.0381 | **1.625** | 0.978 | 0.74 | 0.698 |
| 172 | FLIWSITFLK | 21 | 30 | 10 | 0.5612 | NA | NA | **0.72** | 0.701 |
| 174 | NNEYK | 23 | 27 | 5 | 0.9634 | **1.184** | NA | 0.7 | 0.725 |
| 193 | YKIVNYPPLEIRLV | 26 | 39 | 14 | 0.9684 | NA | **0.92** | 0.75 | 0.563 |
| 199 | PPPTSVRAR | 1 | 9 | 9 | 1.0498 | **2.839** | 0.998 | 0.67 | 0.645 |
| 200 | PPLGMNNESTLKRN | 22 | 35 | 14 | 0.7509 | 0.964 | **0.998** | 0.8 | 0.595 |
| 201 | RRSDG | 19 | 23 | 5 | 0.5832 | **1.066** | 0.848 | 0.76 | 0.622 |
| 207 | ISGRCVNAMTTLFF | 2 | 15 | 14 | 0.5313 | 0.851 | **0.935** | 0.68 | 0.72 |
| 212 | EGKLRERKNLWAWS | 27 | 40 | 14 | 0.6181 | NA | **0.773** | 0.71 | 0.69 |
| 215 | RPYSLQPHSQ | 10 | 19 | 10 | 0.5749 | **1.058** | 0.995 | 0.75 | 0.667 |
| 225 | CSQPADLS | 1 | 8 | 8 | 0.6106 | **1.442** | 0.983 | 0.83 | 0.628 |
| 231 | KKIKGNYS | 7 | 14 | 8 | 0.5850 | **0.99** | 0.959 | 0.79 | 0.774 |
| 235 | DITLLASYPT | 16 | 25 | 10 | 0.7591 | NA | NA | **0.75** | 0.649 |

| | | | | | | | | | |
|---|---|---|---|---|---|---|---|---|---|
| 239 | GDCPSRHPG | 16 | 24 | 9 | 0.8723 | **1.559** | 0.921 | 0.74 | 0.76 |
| 248 | ARGSGGYRPSLGE | 29 | 41 | 13 | 0.6260 | **1.55** | NA | 0.77 | 0.702 |
| 250 | SPDGDIA | 15 | 21 | 7 | 0.6892 | **1.472** | 0.919 | 0.82 | 0.625 |
| 252 | FQAPSDH | 1 | 7 | 7 | 0.5520 | **1.341** | 0.902 | 0.72 | 0.672 |
| 255 | AGNCSKPSEGPPIRN | 1 | 15 | 15 | 0.5259 | **2.177** | 1 | 0.82 | 0.615 |
| 256 | LSPINNHQTNTINL | 23 | 36 | 14 | 0.5519 | 0.893 | **0.928** | 0.75 | NA |
| 258 | WTPGDLQ | 34 | 40 | 7 | 0.7748 | **1.419** | 0.886 | 0.8 | 0.7 |
| 261 | PCSWHGKTTALGIY | 8 | 21 | 14 | 1.2817 | NA | **0.959** | 0.69 | NA |
| 264 | IRDVHKMR | 1 | 8 | 8 | 0.7260 | NA | NA | 0.63 | **0.753** |
| 265 | QKRWPGGSPGSMLPGAGP KR | 21 | 40 | 20 | 0.6826 | **2.177** | 1 | 0.78 | 0.679 |
| 266 | NEPDD | 1 | 5 | 5 | 0.5234 | **2.59** | NA | 0.69 | NA |
| 272 | FRPDPRKG | 5 | 12 | 8 | 0.7476 | **1.678** | 1 | 0.71 | NA |
| 275 | TARNSKS | 1 | 7 | 7 | 0.7682 | **1.199** | 0.792 | 0.66 | NA |
| 282 | GTGDSH | 8 | 13 | 6 | 0.7646 | **1.664** | NA | 0.67 | 0.645 |
| 285 | SEAEEN | 9 | 14 | 6 | 0.9762 | **1.479** | 0.908 | 0.74 | NA |
| 286 | SNYLTCDRAN | 7 | 16 | 10 | 0.6336 | 0.83 | 0.844 | **0.86** | NA |
| 289 | SYTTNSD | 33 | 39 | 7 | 1.2634 | **1.719** | 0.992 | 0.69 | NA |
| 291 | DSRHSFFYAY | 10 | 19 | 10 | 0.6582 | NA | NA | **0.66** | NA |
| 294 | TCYARGSNCVSGVL | 24 | 37 | 14 | 0.5693 | NA | **0.982** | 0.73 | 0.747 |
| 297 | TGGAMPPLFAWSCN | 3 | 16 | 14 | 1.3689 | NA | **0.992** | 0.72 | 0.769 |
| 302 | VVRLRSVHDRFYHF | 7 | 20 | 14 | 0.7319 | NA | **0.998** | 0.66 | 0.724 |
| 303 | IKNSKGEGKST | 10 | 20 | 11 | 1.0484 | **1.922** | 0.998 | 0.76 | NA |
| 307 | SNPEDAS | 25 | 31 | 7 | 0.6955 | **1.761** | 0.999 | 0.75 | NA |
| 308 | IRNSGEAP | 1 | 8 | 8 | 0.6157 | **1.377** | 0.967 | 0.78 | NA |
| 310 | YKALSNKSTY | 7 | 16 | 10 | 0.5905 | NA | 0.706 | **0.73** | NA |
| 311 | SHSKSISDTDRH | 25 | 36 | 12 | 0.9743 | **1.101** | NA | 0.65 | NA |
| 314 | SVNITGFSQNFIKY | 2 | 15 | 14 | 0.5950 | NA | **0.751** | 0.7 | NA |
| 328 | RYHEGK | 33 | 38 | 6 | 0.9658 | NA | 0.773 | 0.59 | **0.825** |
| 329 | ISRLYLCFDL | 11 | 20 | 10 | 0.7175 | NA | | **0.73** | NA |
| 330 | CGLTIAPVSC | 5 | 14 | 10 | 0.5093 | NA | 0.752 | **0.77** | NA |
| 332 | RKEAHNKEQ | 30 | 38 | 9 | 0.6059 | **1.31** | 0.896 | 0.72 | 0.695 |
| 334 | PNNVGG | 32 | 37 | 6 | 0.5350 | **1.518** | 1 | 0.69 | NA |
| 336 | DDIR | 28 | 31 | 4 | 0.7686 | **1.113** | NA | 0.73 | 0.605 |
| 338 | KSHFFRCDIR | 18 | 27 | 10 | 0.8699 | NA | NA | **0.7** | 0.685 |
| 343 | SGVPS | 11 | 15 | 6 | 0.5381 | **1.084** | 0.835 | 0.6 | NA |
| 344 | SQYDSKHEKGSDE | 13 | 25 | 13 | 0.9267 | **1.702** | 0.891 | 0.75 | NA |
| 345 | ECPVG | 3 | 7 | 5 | 0.7878 | **1.073** | NA | 0.83 | NA |
| 349 | FQLTLLLSFT | 17 | 26 | 10 | 0.7393 | NA | NA | **0.59** | NA |
| 354 | RTDGTARE | 4 | 11 | 8 | 1.1388 | **1.333** | 0.982 | 0.77 | NA |
| 358 | GHVHERESRWTRLK | 3 | 17 | 14 | 0.5883 | NA | **0.96** | 0.77 | NA |
| 360 | FHFGPIVGPKRKCQ | 9 | 22 | 14 | 0.9196 | 0.84 | **0.997** | 0.71 | NA |

| | | | | | | | | | |
|---|---|---|---|---|---|---|---|---|---|
| 363 | SSGSP | 1 | 5 | 5 | 1.1179 | **2.063** | 0.992 | 0.66 | 0.788 |
| 364 | RACCLVDVVI | 17 | 26 | 10 | 0.7174 | NA | NA | **0.74** | NA |
| 370 | VPSVSQRVQCTGIR | 2 | 15 | 14 | 0.9746 | NA | **0.684** | 0.67 | NA |
| 373 | SAQNGRLSIN | 7 | 16 | 10 | 0.8620 | NA | NA | **0.75** | NA |
| 377 | ATINQNDSQGPKKNADNV PTF | 16 | 36 | 21 | 1.0211 | **2.28** | 1 | 0.8 | NA |
| 379 | PLYFRGQRSR | 15 | 24 | 10 | 0.8754 | NA | NA | **0.77** | 0.714 |
| 383 | NGEKRC | 31 | 36 | 6 | 0.6413 | **0.941** | NA | 0.76 | NA |
| 385 | SNSPQA | 9 | 14 | 6 | 0.9659 | **1.086** | 0.942 | 0.67 | 0.599 |
| 396 | WPFLIAGQCI | 11 | 20 | 10 | 0.8620 | NA | NA | **0.69** | 0.569 |
| 403 | KNPPTPSRSYPG | 25 | 36 | 12 | 0.7773 | **2.575** | 0.997 | 0.7 | 0.577 |
| 405 | CNFPAGAKKLKGAS | 10 | 23 | 14 | 0.7190 | 0.92 | **0.999** | 0.82 | 0.514 |
| 411 | NSQNQKSI | 28 | 35 | 8 | 0.8313 | **1.32** | 0.99 | 0.82 | 0.603 |
| 413 | GFEA | 15 | 18 | 4 | 1.0085 | **0.841** | 0.737 | 0.68 | NA |
| 422 | IRPRPPPPN | 5 | 13 | 9 | 0.5049 | **2.298** | 1 | 0.84 | 0.7 |
| 424 | LRYTVARFNYTTGM | 23 | 36 | 14 | 0.7104 | NA | **0.942** | 0.74 | NA |
| 427 | YESVIQVNVTINHC | 15 | 28 | 14 | 1.0655 | NA | **0.874** | 0.63 | NA |
| 429 | ARSSTRTGFQPSKD | 1 | 14 | 14 | 1.0654 | **1.406** | 0.997 | 0.69 | NA |
| 432 | SSYSPEEGEYG | 1 | 11 | 11 | 1.0349 | **1.982** | 1 | 0.8 | 0.611 |
| 433 | GEGKSC | 1 | 6 | 6 | 0.7630 | **1.701** | 0.838 | 0.82 | 0.683 |
| 452 | DTATTQEIS | 27 | 35 | 9 | 0.5742 | **1.125** | 0.973 | 0.69 | 0.681 |
| 453 | EWKNEGA | 1 | 7 | 7 | 0.5007 | **1.24** | 0.864 | 0.76 | 0.662 |
| 454 | IRPASIP | 20 | 26 | 7 | 0.6507 | **1.084** | 0.999 | 0.74 | 0.527 |
| 456 | LVPCFYCLKPPRPL | 11 | 24 | 14 | 0.6325 | NA | **0.986** | 0.75 | 0.624 |
| 462 | IQAAF | 31 | 35 | 5 | 0.9774 | NA | NA | 0.78 | **0.783** |
| 464 | KQEGDARENALPREE | 21 | 35 | 15 | 0.9804 | **1.733** | 0.983 | 0.76 | 0.786 |
| 469 | NDTRLRYSYR | 11 | 20 | 10 | 1.0627 | NA | NA | **0.71** | 0.64 |
| 477 | IVACLTVQLK | 6 | 15 | 10 | 0.7938 | NA | NA | **0.78** | 0.586 |
| 484 | DAWEGDTKRK | 25 | 34 | 10 | 0.7485 | **1.448** | 0.998 | 0.56 | 0.836 |
| 489 | EGVTTSCRHKKTTR | 5 | 18 | 14 | 0.9239 | NA | **0.959** | 0.77 | 0.743 |
| 494 | SYFH | 1 | 4 | 4 | 0.8404 | NA | NA | 0.71 | **0.814** |
| 508 | PMLPLFARPPLDGL | 20 | 33 | 14 | 0.6352 | NA | **0.953** | 0.67 | 0.618 |
| 510 | FPEENRGDTAPAPKDWQS G | 16 | 34 | 9 | 0.5527 | **2.369** | 1 | 0.84 | 0.627 |
| 512 | DKRDSNENS | 10 | 18 | 9 | 0.7346 | **1.757** | 1 | 0.64 | 0.662 |
| 516 | AEPASFSLRFYSHL | 19 | 32 | 14 | 0.5579 | NA | **0.828** | 0.63 | 0.642 |
| 524 | QENHSS | 17 | 22 | 6 | 0.6572 | **1.836** | 0.859 | 0.73 | 0.614 |
| 526 | VKMTQPQQ | 27 | 34 | 8 | 0.9036 | **1.941** | 0.734 | 0.68 | NA |
| 529 | SQVPQGEKT | 26 | 34 | 9 | 0.6204 | **2.039** | 0.921 | 0.81 | 0.607 |
| 536 | FKPQTKGVT | 26 | 34 | 9 | 0.5033 | **1.289** | 0.747 | 0.73 | 0.549 |
| 541 | TSRNVSMRAKAYCD | 3 | 16 | 14 | 0.7015 | 0.703 | **0.98** | 0.84 | 0.76 |
| 556 | ITDVYFQNNLFESQ | 5 | 18 | 14 | 0.5580 | NA | **0.849** | 0.69 | 0.612 |
| 559 | GYKERPNKNTVA | 13 | 24 | 12 | 0.7165 | **1.538** | 0.987 | 0.85 | 0.637 |

| | | | | | | | | | |
|---|---|---|---|---|---|---|---|---|---|
| 564 | DKGVG | 30 | 34 | 5 | 1.0503 | **1.744** | 1 | 0.76 | 0.632 |
| 567 | ACAEQKP | 6 | 12 | 7 | 0.6613 | **1.069** | 0.979 | 0.7 | 0.706 |
| 572 | DLGISEEKSLCRQA | 20 | 33 | 14 | 0.8322 | NA | **0.739** | 0.68 | NA |
| 577 | QRDNPTR | 13 | 19 | 7 | 0.6135 | **1.304** | 0.969 | 0.74 | 0.693 |
| 579 | KSPSSGGRANKGNI | 1 | 14 | 14 | 1.1311 | **2.387** | 0.999 | 0.72 | 0.64 |
| 584 | PYFLKNANIMSPSS | 16 | 29 | 14 | 0.5788 | 0.8 | **0.941** | 0.84 | 0.628 |
| 591 | PPVT | 26 | 29 | 4 | 0.5700 | **0.878** | 0.686 | 0.66 | 0.606 |
| 596 | YDGESPRRL | 24 | 32 | 9 | 0.7385 | **1.287** | 0.996 | 0.75 | 0.721 |
| 604 | ASPLPGSWSGN | 2 | 12 | 11 | 0.5647 | **1.675** | 0.999 | 0.68 | 0.612 |
| 605 | IGNEKKTKHEVR | 7 | 18 | 12 | 1.1770 | **1.221** | 0.99 | 0.83 | 0.717 |
| 609 | ECRAGRD | 27 | 33 | 7 | 1.1808 | **1.054** | NA | 0.67 | 0.712 |
| 610 | AGEKGKGECG | 13 | 22 | 10 | 1.1666 | **1.455** | NA | 0.64 | 0.621 |
| 613 | TLYLWFKSSRSCPI | 9 | 22 | 14 | 0.7107 | NA | **0.855** | 0.71 | 0.782 |
| 621 | LYKYESCTGEIVSI | 5 | 18 | 14 | 0.8371 | NA | **0.926** | 0.68 | 0.727 |
| 623 | GAPAREEN | 1 | 8 | 8 | 1.3326 | **1.814** | NA | 0.73 | 0.707 |
| 629 | CKEGGVP | 27 | 33 | 7 | 1.1277 | **1.46** | 0.707 | 0.65 | 0.697 |
| 631 | EYLR | 30 | 33 | 4 | 0.6754 | NA | 0.802 | 0.77 | **0.864** |
| 633 | VKNAWIPVVSVCVV | 8 | 21 | 14 | 0.7406 | NA | **0.946** | 0.61 | 0.559 |
| 634 | DEDHRQGEKI | 24 | 33 | 10 | 0.9834 | **1.578** | 0.988 | 0.62 | 0.727 |
| 635 | ISRGGSRKS | 2 | 9 | 8 | 1.3082 | **1.437** | NA | 0.73 | 0.646 |
| 636 | FLAR | 1 | 4 | 4 | 0.5119 | NA | NA | 0.65 | **0.75** |
| 640 | RSSRAH | 1 | 6 | 6 | 0.6838 | NA | 0.693 | 0.66 | **0.717** |
| 643 | GRDKGE | 28 | 33 | 6 | 0.7836 | **1.898** | 0.737 | 0.54 | 0.818 |
| 646 | SELT | 20 | 23 | 4 | 0.5318 | **1.043** | 0.976 | 0.55 | 0.594 |
| 655 | SRMTN | 27 | 31 | 5 | 0.5349 | **0.957** | NA | 0.69 | 0.63 |
| 665 | NGIKINQRITFKSN | 10 | 23 | 14 | 0.6472 | NA | **0.935** | 0.75 | 0.602 |
| 666 | YDVDYFTIPIKPVS | 12 | 25 | 14 | 0.7780 | NA | **0.993** | 0.71 | 0.594 |
| 668 | IADKKGSRAKKGPTF | 1 | 15 | 15 | 1.4644 | **1.593** | 0.933 | 0.82 | 0.693 |
| 674 | IPETKYKQQEPAEQ | 1 | 14 | 14 | 0.9184 | **1.643** | 0.998 | 0.83 | 0.672 |
| 676 | SPEQAP | 8 | 13 | 6 | 1.3297 | **1.424** | 0.95 | 0.73 | 0.641 |
| 677 | RKPSRKS | 18 | 24 | 7 | 0.6659 | **1.202** | NA | 0.8 | 0.805 |
| 679 | ITQKH | 1 | 5 | 5 | 0.5326 | NA | NA | 0.63 | **0.706** |
| 686 | ELIQIIPNDLSSSI | 10 | 23 | 14 | 0.6341 | NA | **0.982** | 0.66 | 0.805 |
| 696 | TIRTLEIRIV | 1 | 10 | 10 | 0.9326 | NA | NA | **0.74** | 0.698 |
| 701 | ILRGPCSEYPFNLI | 1 | 14 | 14 | 0.6818 | NA | **0.933** | 0.78 | 0.541 |
| 702 | FQLLY | 28 | 32 | 5 | 0.6788 | NA | 0.78 | 0.69 | **0.781** |
| 716 | IKKA | 1 | 4 | 4 | 0.6917 | NA | NA | 0.62 | **0.805** |
| 724 | PVIK | 7 | 10 | 4 | 0.9933 | **0.914** | 0.792 | 0.66 | 0.716 |
| 725 | SGKEKGNPH | 9 | 17 | 9 | 0.8070 | **1.833** | 0.968 | 0.74 | 0.538 |
| 733 | DIFSECSLKY | 13 | 22 | 10 | 0.9328 | NA | NA | **0.81** | 0.782 |
| 735 | KGPPI | 9 | 13 | 5 | 0.8213 | **1.222** | NA | 0.59 | 0.584 |

| | | | | | | | | | |
|---|---|---|---|---|---|---|---|---|---|
| 737 | KREQDHL | 1 | 7 | 7 | 1.1429 | NA | NA | 0.59 | **0.654** |
| 739 | VLPGRYLRPDLLLI | 5 | 18 | 14 | 0.6380 | NA | **0.876** | 0.75 | 0.584 |
| 743 | TKRTAGQT | 24 | 31 | 8 | 0.8439 | **1.413** | 0.764 | 0.72 | 0.79 |
| 745 | TRDHQ | 1 | 5 | 5 | 0.5829 | **1.133** | NA | 0.78 | 0.787 |
| 754 | SQQRS | 8 | 12 | 5 | 0.6915 | **1.225** | 0.839 | 0.69 | NA |
| 756 | SQFPN | 27 | 31 | 5 | 0.5754 | **1.239** | 0.966 | 0.75 | 0.585 |
| 763 | VASQGARPAPSAKA | 18 | 31 | 14 | 0.8840 | **1.966** | 0.887 | 0.69 | 0.71 |
| 767 | RHNGSNE | 25 | 31 | 7 | 1.1731 | **1.82** | 0.952 | 0.74 | 0.658 |
| 768 | IYPPPHQ | 25 | 31 | 7 | 0.6819 | **2.437** | 0.939 | 0.67 | 0.671 |
| 769 | KCCQKAYCQR | 5 | 14 | 10 | 0.6367 | NA | NA | **0.81** | 0.79 |
| 774 | TLHRINNPDQ | 5 | 14 | 10 | 0.5385 | NA | NA | **0.65** | 0.639 |
| 776 | NHVKHASDAKVVQE | 17 | 30 | 14 | 0.5382 | NA | **0.935** | 0.7 | NA |
| 777 | KSGYMGAKSLTITP | 1 | 14 | 14 | 1.0021 | 0.656 | **0.91** | 0.69 | 0.753 |
| 778 | SSPSSA | 1 | 6 | 6 | 0.7916 | **2.228** | 0.888 | 0.76 | 0.794 |
| 785 | QGAEIPGP | 15 | 22 | 8 | 0.5532 | **1.512** | 1 | 0.76 | 0.566 |
| 789 | PGEPGQM | 25 | 31 | 7 | 1.1405 | **2.168** | 0.962 | 0.65 | 0.667 |
| 792 | LPEGDSAAT | 22 | 30 | 9 | 0.7242 | **1.352** | 0.936 | 0.68 | 0.585 |
| 795 | QNIASARQKK | 22 | 31 | 10 | 0.6396 | **0.968** | 0.886 | 0.65 | 0.624 |
| 798 | TYNG | 9 | 12 | 4 | 0.7300 | **1.056** | 0.995 | 0.85 | 0.694 |
| 807 | YPIHTHNKAI | 11 | 20 | 10 | 0.6854 | NA | NA | **0.78** | 0.75 |
| 809 | NMVFTFLTEQ | 12 | 21 | 10 | 0.6750 | NA | NA | **0.68** | 0.625 |
| 811 | FNPSLLLPCPCRRC | 2 | 15 | 14 | 0.5480 | 0.921 | **0.989** | 0.72 | 0.783 |
| 812 | PFSTFRLRTC | 9 | 18 | 10 | 0.7886 | NA | NA | **0.63** | 0.547 |
| 815 | LNSLPAQITF | 14 | 23 | 10 | 0.7563 | NA | NA | **0.75** | **0.75** |
| 818 | QQIRPEIA | 8 | 15 | 8 | 0.8274 | **0.851** | NA | 0.83 | 0.7 |
| 822 | QGSTRNGKR | 14 | 22 | 9 | 1.1369 | **1.428** | 0.72 | 0.8 | 0.683 |
| 828 | THLTDSCNKSFKFC | 13 | 26 | 14 | 0.5253 | NA | **0.812** | 0.7 | 0.575 |
| 830 | PTGF | 1 | 4 | 4 | 0.9847 | NA | NA | 0.57 | **0.767** |
| 835 | NFNQRVYCYN | 4 | 13 | 10 | 0.6580 | NA | NA | **0.81** | 0.725 |
| 837 | AARQDDEQKN | 21 | 30 | 10 | 1.1541 | **2.064** | NA | 0.74 | 0.647 |
| 841 | QGNGEGNANH | 5 | 14 | 10 | 0.8442 | **1.9** | 0.999 | 0.82 | 0.667 |
| 842 | PVPHRASLSGG | 1 | 11 | 11 | 0.9334 | **1.237** | NA | 0.7 | NA |
| 843 | HNADSKRS | 8 | 15 | 8 | 1.1758 | **1.19** | 0.957 | 0.81 | 0.638 |
| 844 | GLSPDEIGDT | 12 | 21 | 10 | 0.7774 | **1.742** | 0.982 | 0.77 | 0.692 |
| 850 | TIESLRFRLQ | 12 | 21 | 10 | 0.9407 | NA | NA | **0.67** | 0.557 |
| 854 | LPLRLPSTNTRVNL | 6 | 19 | 14 | 1.2511 | 0.673 | **0.991** | 0.72 | 0.667 |
| 857 | NTEGRIISWPCWSL | 7 | 20 | 14 | 0.9152 | NA | **0.978** | 0.71 | 0.56 |
| 858 | QFNDSSTDFEY | 1 | 11 | 11 | 0.8875 | **1.239** | 0.998 | 0.69 | 0.539 |
| 859 | KASREERPSPDDKFK | 1 | 15 | 15 | 0.9000 | **2.493** | 0.999 | 0.68 | 0.567 |
| 860 | TPPYTFLFLKCTAG | 1 | 14 | 14 | 0.5292 | NA | **0.909** | 0.67 | 0.7 |
| 865 | ACCTLKKQEGVRRR | 17 | 30 | 14 | 0.8532 | NA | **0.992** | 0.71 | 0.671 |

| | | | | | | | | | |
|---|---|---|---|---|---|---|---|---|---|
| 866 | HEKTDA | 19 | 24 | 6 | 0.6405 | **0.97** | NA | 0.72 | 0.66 |
| 867 | ARQNSPRNV | 18 | 26 | 9 | 0.5485 | **1.62** | 0.977 | 0.7 | 0.7 |
| 868 | SPSPVASA | 8 | 15 | 8 | 0.7463 | **1.521** | NA | 0.74 | 0.558 |
| 869 | GLRRESRSAG | 13 | 22 | 10 | 0.9408 | **1.041** | 0.972 | 0.72 | 0.527 |
| 871 | VDFLVFIMCG | 9 | 18 | 10 | 1.0651 | NA | NA | **0.79** | 0.559 |
| 879 | IQPKLY | 12 | 17 | 6 | 0.9030 | 0.668 | NA | 0.65 | **0.711** |
| 880 | PEVTLSPVKI | 8 | 17 | 10 | 0.6948 | NA | NA | **0.7** | 0.572 |
| 884 | RSQCGESRCHENSD | 7 | 20 | 14 | 1.1797 | **1.097** | 0.999 | 0.78 | 0.708 |
| 885 | GEGMGEKGGTNG | 19 | 30 | 12 | 1.2637 | **2.034** | 0.999 | 0.69 | 0.633 |
| 886 | VYNACAGHYHPPLR | 13 | 26 | 14 | 0.8348 | NA | **0.827** | 0.74 | 0.594 |
| 890 | SRCEM | 1 | 5 | 5 | 0.8630 | NA | NA | 0.63 | **0.767** |
| 899 | RLNFTA | 25 | 30 | 6 | 0.9318 | NA | NA | 0.76 | **0.789** |
| 900 | PLLISSAVIR | 2 | 11 | 10 | 0.9266 | NA | NA | **0.66** | NA |
| 901 | PCQEY | 25 | 29 | 5 | 0.6774 | **0.8** | 0.771 | 0.59 | 0.7 |
| 907 | KACRVDGRHH | 3 | 12 | 10 | 0.6575 | NA | NA | **0.84** | 0.687 |
| 909 | RRGGRH | 17 | 22 | 6 | 0.5577 | **1.304** | NA | 0.74 | 0.621 |
| 910 | SGERS | 26 | 30 | 5 | 1.2411 | **1.42** | NA | 0.64 | 0.642 |
| 912 | LQEGRA | 25 | 30 | 6 | 0.6979 | **1.181** | 0.929 | 0.84 | 0.692 |

## Table S2 (a): Epitopes of *Synpeps* binding to MHC Class I Allele HLA-A*0201

| **Allele:HLA-A*0201** | | | | |
|---|---|---|---|---|
| **PSP ID** | **Peptide** | **Position** | **Affinity IC50 (nM)** | **logscore** |
| 1 | FLWESSTYA | 2 | 6 | 0.827 |
| | TMVSILFLI | 34 | 10 | 0.782 |
| | FLIKILPNM | 40 | 12 | 0.767 |
| | ALCFAMPAV | 10 | 22 | 0.712 |
| | ILPNMLAIV | 44 | 31 | 0.682 |
| | NMLAIVSCI | 47 | 46 | 0.645 |
| 5 | AQSSSLWGV | 26 | 13 | 0.761 |
| | SLWGVAPFA | 30 | 14 | 0.754 |
| 26 | ILNSNNWLL | 28 | 15 | 0.747 |
| | HISGYLIPI | 0 | 42 | 0.654 |
| | FILNSNNWL | 27 | 47 | 0.643 |
| 33 | IVGHIIFYV | 7 | 15 | 0.747 |
| 35 | ILFPLFRPI | 18 | 32 | 0.678 |
| 108 | FLTAPPSPA | 7 | 39 | 0.661 |

| | | | | |
|---|---|---|---|---|
| 119 | SLMLLMSYA | 19 | 17 | 0.733 |
| 142 | IILAHLLLV | 6 | 12 | 0.767 |
| 150 | SIYFCLHFI | 9 | 23 | 0.706 |
| 155 | ILSDSSESL | 13 | 38 | 0.662 |
| | FLIWSITFL | 20 | 6 | 0.821 |
| | SLINSCFKL | 5 | 14 | 0.75 |
| 172 | ALLMRHFLI | 14 | 32 | 0.678 |
| | FMLLCANLL | 3 | 12 | 0.765 |
| 174 | LLNIMVAFL | 10 | 50 | 0.638 |
| 207 | SLLPGNYTV | 27 | 8 | 0.804 |
| 264 | ALLFFILHV | 15 | 9 | 0.791 |
| | YIYFCTLLL | 18 | 10 | 0.78 |
| | LLLHCVITL | 24 | 18 | 0.73 |
| 291 | LLHCVITLL | 25 | 34 | 0.674 |
| | FLNFLFYFL | 24 | 8 | 0.8 |
| 297 | FLFYFLFLF | 27 | 19 | 0.724 |
| | ALLSSIFRL | 18 | 9 | 0.796 |
| | LLSEGYYKA | 0 | 28 | 0.69 |
| 310 | FLFKLVSMT | 27 | 37 | 0.666 |
| 349 | RLVYVAFLV | 28 | 14 | 0.752 |
| | FTLLRLVYV | 24 | 16 | 0.744 |
| 355 | FLLKFTGGL | 28 | 18 | 0.729 |
| 363 | NTLTGIFTV | 20 | 38 | 0.663 |
| 371 | FLVVKQYLA | 2 | 27 | 0.693 |
| | YLARSNCKI | 8 | 49 | 0.639 |
| 373 | IMMSDQNYM | 28 | 21 | 0.716 |
| 376 | YISGRIDFL | 7 | 42 | 0.653 |
| 385 | TLFRWPFLI | 14 | 9 | 0.791 |
| 424 | YMLICCCVL | 10 | 15 | 0.748 |
| 495 | ALISLLQSV | 26 | 10 | 0.782 |
| 516 | YLLSVVILA | 10 | 10 | 0.786 |
| | SLFKKRFYL | 3 | 16 | 0.741 |
| 536 | LLQAANFSL | 4 | 14 | 0.754 |
| | SLIFKLFDV | 21 | 11 | 0.777 |
| 539 | KLFDVYKPI | 25 | 13 | 0.76 |
| | LLSSENLPI | 12 | 42 | 0.654 |
| 619 | LLSRCFTFL | 0 | 25 | 0.701 |
| 623 | KIFLCMPIV | 22 | 16 | 0.739 |

| | | | | |
|---|---|---|---|---|
| 629 | LIAAFLLRL | 12 | 47 | 0.644 |
| 778 | FLARINAQL | 13 | 18 | 0.733 |
| 787 | KTYEILAGL | 12 | 20 | 0.721 |
| 804 | TLLPETALI | 14 | 32 | 0.678 |
| 833 | FVSYLYFKV | 14 | 12 | 0.767 |
| 905 | SLYRENETV | 5 | 44 | 0.649 |

## Table S2 (b): Epitopes of *Synpeps* binding to MHC Class I Allele HLA-B*1501

| Allele:HLA-B*1501 | | | | | |
|---|---|---|---|---|---|
| **Eka Protein ID** | **Peptide** | **Position** | **Affinity IC50(nM)** | | **Logscore** |
| | LLKRSNIHY | 61 | 36 | SB | 0.667 |
| 1 | TIHAANLHF | 24 | 49 | SB | 0.639 |
| | LIYSFNQHF | 16 | 28 | SB | 0.691 |
| 26 | RLRFLIYSF | 12 | 34 | SB | 0.672 |
| | YTFALSISF | 42 | 24 | SB | 0.704 |
| | RQIVGHIIF | 5 | 41 | SB | 0.655 |
| 33 | SSSRCFAPY | 18 | 43 | SB | 0.651 |
| 35 | ASRIYFCSF | 39 | 29 | SB | 0.688 |
| 71 | LLSELGLEF | 28 | 49 | SB | 0.64 |
| 119 | YIIQAMNYF | 5 | 43 | SB | 0.651 |
| 150 | FSIYFCLHF | 8 | 33 | SB | 0.675 |
| 155 | SISSHSRAF | 22 | 37 | SB | 0.664 |
| 174 | RIRFQFLSY | 30 | 34 | SB | 0.672 |
| 193 | SLSSYRLHY | 5 | 35 | SB | 0.67 |
| 289 | ITINLTTSY | 25 | 40 | SB | 0.657 |
| 291 | SQQDSRHSF | 6 | 32 | SB | 0.679 |
| 297 | YIAGHFLNF | 19 | 41 | SB | 0.656 |
| 349 | FQLTLLLSF | 16 | 39 | SB | 0.661 |
| 385 | FLIMPVTVF | 20 | 23 | SB | 0.708 |
| 433 | YMYYLVTSY | 25 | 21 | SB | 0.715 |
| 465 | ILCCTSQPM | 24 | 46 | SB | 0.645 |
| 536 | FLFSNALPY | 16 | 28 | SB | 0.691 |
| 539 | LIFKLFDVY | 22 | 46 | SB | 0.644 |
| 619 | RIIKSKGSF | 15 | 48 | SB | 0.642 |
| 629 | FIFCLIAAF | 8 | 29 | SB | 0.686 |
| 778 | ALPFSFLPF | 5 | 34 | SB | 0.673 |

| | | | | |
|---|---|---|---|---|
| 787 | GLKRHTTVF | 19 | 31 SB | 0.681 |
| | RLHESGAFF | 0 | 38 SB | 0.663 |
| 833 | SARAFFVSY | 9 | 49 SB | 0.639 |
| 905 | FLFSRFTDY | 18 | 40 SB | 0.659 |

## Table S2 (c): Epitopes of *Synpeps* binding to MHC Class I Allele HLA-B*2705

| Allele:HLA-B*2705 | | | | |
|---|---|---|---|---|
| **Eka Protein ID** | **Peptide** | **Position** | **Affinity IC50(nM)** | **logscore** |
| 5 | RRSYLVYMA | 52 | 48 SB | 0.642 |
| 35 | RRILFPLFR | 16 | 19 SB | 0.724 |
| | RRRILFPLF | 15 | 32 SB | 0.677 |
| | LRRRILFPL | 14 | 34 SB | 0.674 |
| 108 | ARMNILLAI | 16 | 17 SB | 0.737 |
| 142 | ARIILAHLL | 4 | 23 SB | 0.707 |
| 172 | MRHFLIWSI | 17 | 37 SB | 0.664 |
| 193 | YRLHYILRV | 9 | 25 SB | 0.7 |
| 207 | MRLSIFSLL | 21 | 30 SB | 0.683 |
| 264 | ARALLFFIL | 13 | 30 SB | 0.683 |
| | HRVRVARAL | 8 | 47 SB | 0.644 |
| 289 | FRTNNGTKM | 11 | 32 SB | 0.678 |
| 297 | LRYIAGHFL | 17 | 49 SB | 0.639 |
| 310 | YRHALLSSI | 15 | 38 SB | 0.663 |
| 349 | LRLVYYVAFL | 27 | 48 SB | 0.642 |
| 355 | HRRCLLFLL | 22 | 24 SB | 0.705 |
| | RRCLLFLLK | 23 | 28 SB | 0.69 |
| 363 | YRYMFLKQL | 5 | 25 SB | 0.701 |
| 371 | SRYRKRLAL | 23 | 42 SB | 0.653 |
| 373 | GRLSINAHL | 10 | 42 SB | 0.654 |
| 376 | GRIDFLVTM | 10 | 39 SB | 0.661 |
| 424 | ARFNYTTGM | 27 | 16 SB | 0.739 |
| 433 | YRTYMYYLV | 22 | 46 SB | 0.646 |
| 465 | SRFNTLTQI | 10 | 36 SB | 0.667 |
| 495 | RRLSVLARR | 0 | 21 SB | 0.715 |
| 516 | KRFYLLSVV | 7 | 32 SB | 0.679 |
| 623 | IRLKIFLCM | 19 | 26 SB | 0.698 |
| 804 | RRGFYLTLL | 8 | 45 SB | 0.647 |

## Table S2 (d): Epitopes of *Synpeps* binding to MHC Class II Allele HLA-DRB10101

| Allele: HLA-DRB10101 | | | |
|---|---|---|---|
| Eka Protein ID | Peptide | Position | Affinity(IC50) |
| 1 | SSTYALCFAMPAVSF | 6 | 4.1 |
| | STYALCFAMPAVSFC | 7 | 4.4 |
| | TYALCFAMPAVSFCC | 8 | 4.8 |
| | ESSTYALCFAMPAVS | 5 | 5 |
| | YALCFAMPAVSFCCK | 9 | 5.3 |
| | LFLIKILPNMLAIVS | 39 | 5.4 |
| | ILFLIKILPNMLAIV | 38 | 5.8 |
| | WESSTYALCFAMPAV | 4 | 5.8 |
| | SILFLIKILPNMLAI | 37 | 6.3 |
| | WCFLWESSTYALCFA | 0 | 6.5 |
| | FLIKILPNMLAIVSC | 40 | 6.8 |
| | VSILFLIKILPNMLA | 36 | 7 |
| | LWESSTYALCFAMPA | 3 | 7.5 |
| | CFLWESSTYALCFAM | 1 | 8.6 |
| | AANLHFTTMVSILFL | 27 | 9.2 |
| | LIKILPNMLAIVSCI | 41 | 9.7 |
| | ANLHFTTMVSILFLI | 28 | 10.3 |
| | NLHFTTMVSILFLIK | 29 | 11.1 |
| | FLWESSTYALCFAMP | 2 | 11.3 |
| | GPPYFLLKRSNIHYS | 56 | 11.4 |
| | PPYFLLKRSNIHYSV | 57 | 13.5 |
| | IKILPNMLAIVSCIG | 42 | 13.6 |
| | HAANLHFTTMVSILF | 26 | 14 |
| | MLAIVSCIGPPYFLL | 48 | 14.3 |
| | LHFTTMVSILFLIKI | 30 | 16.2 |
| | ALCFAMPAVSFCCKT | 10 | 16.7 |
| | IGPPYFLLKRSNIHY | 55 | 17.1 |
| | LAIVSCIGPPYFLLK | 49 | 17.9 |
| | PYFLLKRSNIHYSVR | 58 | 18.3 |
| | SFCCKTIHAANLHFT | 19 | 18.3 |
| | YFLLKRSNIHYSVRI | 59 | 18.8 |
| | CKTIHAANLHFTTMV | 22 | 21.8 |
| | VSFCCKTIHAANLHF | 18 | 22.8 |
| | IHYSVRISTQASAFF | 67 | 24.7 |

| | | | |
|---|---|---|---|
| | LCFAMPAVSFCCKTI | 11 | 25.5 |
| | FCCKTIHAANLHFTT | 20 | 27.5 |
| | SVRISTQASAFFVIV | 70 | 28.7 |
| | YSVRISTQASAFFVI | 69 | 29.6 |
| | HYSVRISTQASAFFV | 68 | 31.4 |
| | AVSFCCKTIHAANLH | 17 | 32.1 |
| | VRISTQASAFFVIVY | 71 | 32.2 |
| | HFTTMVSILFLIKIL | 31 | 33.8 |
| | NIHYSVRISTQASAF | 66 | 34.4 |
| | KTIHAANLHFTTMVS | 23 | 36.2 |
| | CCKTIHAANLHFTTM | 21 | 36.2 |
| | AIVSCIGPPYFLLKR | 50 | 37.3 |
| | NMLAIVSCIGPPYFL | 47 | 38.5 |
| | CIGPPYFLLKRSNIH | 54 | 39.7 |
| | MVSILFLIKILPNML | 35 | 45.6 |
| | CFAMPAVSFCCKTIH | 12 | 46.8 |
| 5 | LGQFHCMTAQSSSLW | 18 | 4.2 |
| | GQFHCMTAQSSSLWG | 19 | 4.9 |
| | TLGQFHCMTAQSSSL | 17 | 4.9 |
| | QFHCMTAQSSSLWGV | 20 | 5.2 |
| | FHCMTAQSSSLWGVA | 21 | 6.2 |
| | LLSFSPFRGKTLGQF | 7 | 6.7 |
| | KTLGQFHCMTAQSSS | 16 | 7.4 |
| | ALLSFSPFRGKTLGQ | 6 | 9.4 |
| | GKTLGQFHCMTAQSS | 15 | 10.5 |
| | LSFSPFRGKTLGQFH | 8 | 10.5 |
| | SALLSFSPFRGKTLG | 5 | 12.5 |
| | NSALLSFSPFRGKTL | 4 | 15 |
| | HCMTAQSSSLWGVAP | 22 | 19.4 |
| | FATYHLSINRKCRCI | 37 | 19.4 |
| | SFSPFRGKTLGQFHC | 9 | 21.5 |
| | CMTAQSSSLWGVAPF | 23 | 37.2 |
| | FSPFRGKTLGQFHCM | 10 | 40.2 |
| | RCIRRSYLVYMASSN | 49 | 44.1 |
| | QSSSLWGVAPFATYH | 27 | 45.8 |
| | SSSLWGVAPFATYHL | 28 | 46.4 |
| | PFATYHLSINRKCRC | 36 | 47.1 |
| 26 | FTQFILNSNNWLLRL | 24 | 7.8 |
| | TQFILNSNNWLLRLT | 25 | 8.1 |

| | | | |
|---|---|---|---|
| | SGYLIPILPPRLRFL | 2 | 9.3 |
| | ISGYLIPILPPRLRF | 1 | 11.1 |
| | NNWLLRLTSCFVFFS | 32 | 11.8 |
| | QFILNSNNWLLRLTS | 26 | 11.9 |
| | LRFLIYSFNQHFTQF | 13 | 12.6 |
| | HFTQFILNSNNWLLR | 23 | 12.7 |
| | GYLIPILPPRLRFLI | 3 | 13.3 |
| | HISGYLIPILPPRLR | 0 | 13.4 |
| | SNNWLLRLTSCFVFF | 31 | 13.4 |
| | NSNNWLLRLTSCFVF | 30 | 17 |
| | IYSFNQHFTQFILNS | 17 | 17.2 |
| | NWLLRLTSCFVFFSL | 33 | 17.6 |
| | YLIPILPPRLRFLIY | 4 | 20.1 |
| | FILNSNNWLLRLTSC | 27 | 20.4 |
| | RFLIYSFNQHFTQFI | 14 | 20.9 |
| | RLRFLIYSFNQHFTQ | 12 | 22.3 |
| | LNSNNWLLRLTSCFV | 29 | 22.6 |
| | QHFTQFILNSNNWLL | 22 | 23.6 |
| | LIYSFNQHFTQFILN | 16 | 24 |
| | WLLRLTSCFVFFSLP | 34 | 27.6 |
| | FLIYSFNQHFTQFIL | 15 | 28.3 |
| | YSFNQHFTQFILNSN | 18 | 32.1 |
| | LIPILPPRLRFLIYS | 5 | 33.6 |
| | PRLRFLIYSFNQHFT | 11 | 37.1 |
| | TSCFVFFSLPMCACE | 39 | 38.7 |
| | SCFVFFSLPMCACEE | 40 | 41.6 |
| | LTSCFVFFSLPMCAC | 38 | 41.8 |
| | LLRLTSCFVFFSLPM | 35 | 42.4 |
| | CFVFFSLPMCACEEQ | 41 | 49.8 |
| 33 | APYFLIRIVSGALPL | 24 | 6.4 |
| | YFLIRIVSGALPLPH | 26 | 6.7 |
| | PYFLIRIVSGALPLP | 25 | 6.9 |
| | FAPYFLIRIVSGALP | 23 | 9.3 |
| | FLIRIVSGALPLPHV | 27 | 10.1 |
| | PHVYTFALSISFISI | 39 | 10.3 |
| | HVYTFALSISFISIS | 40 | 12.6 |
| | LPHVYTFALSISFIS | 38 | 12.9 |
| | CFAPYFLIRIVSGAL | 22 | 14.2 |
| | PLPHVYTFALSISFI | 37 | 16 |

| | | | |
|---|---|---|---|
| | LIRIVSGALPLPHVY | 28 | 19.5 |
| | LPLPHVYTFALSISF | 36 | 29.5 |
| | IRIVSGALPLPHVYT | 29 | 35.6 |
| | VLSGRQIVGHIIFYV | 1 | 36.8 |
| | RCFAPYFLIRIVSGA | 21 | 44.2 |
| | LVLSGRQIVGHIIFY | 0 | 45.4 |
| | RIVSGALPLPHVYTF | 30 | 47.6 |
| | FPLFRPIFPYPHSIR | 20 | 10 |
| | LFPLFRPIFPYPHSI | 19 | 13.8 |
| | PLFRPIFPYPHSIRC | 21 | 17.1 |
| 35 | ILFPLFRPIFPYPHS | 18 | 20.5 |
| | LFRPIFPYPHSIRCA | 22 | 24.3 |
| | RILFPLFRPIFPYPH | 17 | 31.2 |
| | PHSIRCAPPASRIYF | 30 | 38.5 |
| | SRIYFCSFDIIHINI | 40 | 43.7 |
| | LEFVFTLKLSPFRLS | 34 | 10.9 |
| | GLEFVFTLKLSPFRL | 33 | 13.7 |
| 71 | EFVFTLKLSPFRLSL | 35 | 14.9 |
| | LGLEFVFTLKLSPFR | 32 | 30.6 |
| | YLYITYQDDKLLSEL | 18 | 49 |
| | IDQFLTAPPSPAARM | 4 | 4.5 |
| | QFLTAPPSPAARMNI | 6 | 4.9 |
| | DQFLTAPPSPAARMN | 5 | 5.1 |
| | FLTAPPSPAARMNIL | 7 | 5.5 |
| | IIDQFLTAPPSPAAR | 3 | 5.6 |
| | PIIDQFLTAPPSPAA | 2 | 7.5 |
| 108 | TPIIDQFLTAPPSPA | 1 | 11.6 |
| | RMNILLAITSDTNVA | 17 | 16.8 |
| | AARMNILLAITSDTN | 15 | 18.1 |
| | MNILLAITSDTNVAI | 18 | 21.1 |
| | ARMNILLAITSDTNV | 16 | 21.8 |
| | PAARMNILLAITSDT | 14 | 22.1 |
| | PSPAARMNILLAITS | 12 | 29.2 |
| | SPAARMNILLAITSD | 13 | 29.9 |
| | LYIIYIIQAMNYFCK | 1 | 5.9 |
| | LSSFIRLEMASSPDR | 30 | 6.6 |
| 119 | LMSYADLLSSFIRLE | 23 | 7 |
| | DLYIIYIIQAMNYFC | 0 | 7.1 |
| | YIIYIIQAMNYFCKP | 2 | 7.2 |

| | | | |
|---|---|---|---|
| | LLMSYADLLSSFIRL | 22 | 7.2 |
| | AMNYFCKPSHSLMLL | 9 | 8.9 |
| | MLLMSYADLLSSFIR | 21 | 8.9 |
| | ADLLSSFIRLEMASS | 27 | 9.4 |
| | DLLSSFIRLEMASSP | 28 | 9.8 |
| | LLSSFIRLEMASSPD | 29 | 9.9 |
| | MSYADLLSSFIRLEM | 24 | 9.9 |
| | MNYFCKPSHSLMLLM | 10 | 10.2 |
| | IIYIIQAMNYFCKPS | 3 | 11.2 |
| | LMLLMSYADLLSSFI | 20 | 11.2 |
| | QAMNYFCKPSHSLML | 8 | 12 |
| | SLMLLMSYADLLSSF | 19 | 14.1 |
| | SYADLLSSFIRLEMA | 25 | 15.6 |
| | NYFCKPSHSLMLLMS | 11 | 16 |
| | HSLMLLMSYADLLSS | 18 | 16.3 |
| | SHSLMLLMSYADLLS | 17 | 16.4 |
| | IQAMNYFCKPSHSLM | 7 | 20.3 |
| | IYIIQAMNYFCKPSH | 4 | 20.4 |
| | PSHSLMLLMSYADLL | 16 | 20.7 |
| | YFCKPSHSLMLLMSY | 12 | 22.9 |
| | YADLLSSFIRLEMAS | 26 | 25.6 |
| | IIQAMNYFCKPSHSL | 6 | 35.2 |
| 142 | ILAHLLLVPGIAVKT | 7 | 8.4 |
| | IILAHLLLVPGIAVK | 6 | 8.5 |
| | RIILAHLLLVPGIAV | 5 | 8.6 |
| | LAHLLLVPGIAVKTP | 8 | 9.8 |
| | AHLLLVPGIAVKTPI | 9 | 12.3 |
| | HLLLVPGIAVKTPIT | 10 | 14.4 |
| | ARIILAHLLLVPGIA | 4 | 22.5 |
| | LLLVPGIAVKTPITL | 11 | 25.4 |
| | SFAAARIILAHLLLV | 0 | 26.4 |
| | FAAARIILAHLLLVP | 1 | 40.3 |
| | AARIILAHLLLVPGI | 3 | 42.3 |
| 150 | CLHFIQILNIPSDIL | 13 | 5.3 |
| | LHFIQILNIPSDILK | 14 | 5.7 |
| | FCLHFIQILNIPSDI | 12 | 5.9 |
| | IYFCLHFIQILNIPS | 10 | 6.2 |
| | YFCLHFIQILNIPSD | 11 | 6.3 |
| | HFIQILNIPSDILKE | 15 | 6.8 |

| | | | |
|---|---|---|---|
| | FIQILNIPSDILKEL | 16 | 7.4 |
| | SDILKELSLHSAIFF | 24 | 14.1 |
| | PSDILKELSLHSAIF | 23 | 16.1 |
| | DILKELSLHSAIFFQ | 25 | 22.9 |
| | IQILNIPSDILKELS | 17 | 23.7 |
| | ILKELSLHSAIFFQD | 26 | 27.1 |
| | IPSDILKELSLHSAI | 22 | 28.5 |
| | FSIYFCLHFIQILNI | 8 | 29.8 |
| | SIYFCLHFIQILNIP | 9 | 36.4 |
| | QILNIPSDILKELSL | 18 | 43.8 |
| 155 | SRAFHLLSDSCFSPS | 27 | 29.3 |
| | RAFHLLSDSCFSPSL | 28 | 47.4 |
| 172 | FLKEYVLCGHYLRTS | 27 | 18.1 |
| | TFLKEYVLCGHYLRT | 26 | 21.9 |
| | ITFLKEYVLCGHYLR | 25 | 26.5 |
| | LKEYVLCGHYLRTSN | 28 | 26.5 |
| | SITFLKEYVLCGHYL | 24 | 29.6 |
| | IWSITFLKEYVLCGH | 22 | 34.1 |
| | LIWSITFLKEYVLCG | 21 | 45 |
| | LLMRHFLIWSITFLK | 15 | 48.5 |
| 174 | NIAFMLLCANLLNIM | 0 | 5.7 |
| | IAFMLLCANLLNIMV | 1 | 6.7 |
| | AFMLLCANLLNIMVA | 2 | 8.4 |
| | FMLLCANLLNIMVAF | 3 | 9.8 |
| | DMRIRFQFLSYIGMI | 28 | 17.3 |
| | MLLCANLLNIMVAFL | 4 | 22.9 |
| | HDMRIRFQFLSYIGM | 27 | 25.9 |
| | LLCANLLNIMVAFLY | 5 | 32 |
| | LCANLLNIMVAFLYN | 6 | 49 |
| 193 | HYILRVGKARRGKYK | 12 | 6.5 |
| | YILRVGKARRGKYKI | 13 | 8.1 |
| | LHYILRVGKARRGKY | 11 | 8.9 |
| | RGKYKIVNYPPLEIR | 22 | 9.1 |
| | RLHYILRVGKARRGK | 10 | 10 |
| | YRLHYILRVGKARRG | 9 | 10.3 |
| | RRGKYKIVNYPPLEI | 21 | 12.1 |
| | ILRVGKARRGKYKIV | 14 | 12.8 |
| | GKYKIVNYPPLEIRL | 23 | 13.8 |
| | SYRLHYILRVGKARR | 8 | 15 |

| | | | |
|---|---|---|---|
| | KYKIVNYPPLEIRLV | 24 | 15.8 |
| | ARRGKYKIVNYPPLE | 20 | 18 |
| | KARRGKYKIVNYPPL | 19 | 21.6 |
| | SSYRLHYILRVGKAR | 7 | 22.7 |
| | HPCEISLSSYRLHYI | 0 | 23.8 |
| | LRVGKARRGKYKIVN | 15 | 24.2 |
| | LSSYRLHYILRVGKA | 6 | 29.2 |
| | YKIVNYPPLEIRLVS | 25 | 30.7 |
| | PCEISLSSYRLHYIL | 1 | 36.2 |
| | EISLSSYRLHYILRV | 3 | 44.4 |
| | CEISLSSYRLHYILR | 2 | 49.9 |
| 207 | LSIFSLLPGNYTVPG | 23 | 4.3 |
| | MRLSIFSLLPGNYTV | 21 | 4.4 |
| | RLSIFSLLPGNYTVP | 22 | 4.5 |
| | SIFSLLPGNYTVPGL | 24 | 5 |
| | KMRLSIFSLLPGNYT | 20 | 5.4 |
| | IFSLLPGNYTVPGLS | 25 | 5.7 |
| | FSLLPGNYTVPGLSV | 26 | 7.6 |
| | TLFFESEKSKMRLSI | 11 | 17.6 |
| | LFFESEKSKMRLSIF | 12 | 32.6 |
| | TTLFFESEKSKMRLS | 10 | 39.6 |
| 264 | HKMRHRVRVARALLF | 4 | 9 |
| | KMRHRVRVARALLFF | 5 | 10.3 |
| | RHRVRVARALLFFIL | 7 | 10.3 |
| | MRHRVRVARALLFFI | 6 | 11.4 |
| | FFILHVHLSMTYLCH | 18 | 14.3 |
| | LLFFILHVHLSMTYL | 16 | 14.4 |
| | LFFILHVHLSMTYLC | 17 | 16 |
| | ALLFFILHVHLSMTY | 15 | 16.4 |
| | HRVRVARALLFFILH | 8 | 19.5 |
| | VHKMRHRVRVARALL | 3 | 19.5 |
| | RALLFFILHVHLSMT | 14 | 23.9 |
| | FILHVHLSMTYLCHH | 19 | 24.3 |
| | ARALLFFILHVHLSM | 13 | 28 |
| | VARALLFFILHVHLS | 12 | 32.6 |
| | RVRVARALLFFILHV | 9 | 32.9 |
| | ILHVHLSMTYLCHHQ | 20 | 49.2 |
| 289 | TFSFRTNNGTKMKIE | 8 | 9.9 |
| | LTFSFRTNNGTKMKI | 7 | 11.9 |

| | | | |
|---|---|---|---|
| | FSFRTNNGTKMKIEL | 9 | 14 |
| | NLTFSFRTNNGTKMK | 6 | 25.2 |
| | LKITINLTTSYTTNS | 23 | 27.6 |
| | SFRTNNGTKMKIELK | 10 | 33.1 |
| | KITINLTTSYTTNSD | 24 | 37.2 |
| | KIELKITINLTTSYT | 20 | 44.6 |
| | ELKITINLTTSYTTN | 22 | 49.5 |
| 291 | YIYFCTLLLHCVITL | 18 | 15.9 |
| | AYIYFCTLLLHCVIT | 17 | 19.4 |
| | YAYIYFCTLLLHCVI | 16 | 23 |
| | IYFCTLLLHCVITLL | 19 | 25.6 |
| | FYAYIYFCTLLLHCV | 15 | 28.1 |
| | YFCTLLLHCVITLLM | 20 | 37.3 |
| 297 | CNNLRYIAGHFLNFL | 14 | 9.2 |
| | SCNNLRYIAGHFLNF | 13 | 11.1 |
| | NNLRYIAGHFLNFLF | 15 | 12 |
| | WSCNNLRYIAGHFLN | 12 | 13.5 |
| | AWSCNNLRYIAGHFL | 11 | 15.9 |
| | NLRYIAGHFLNFLFY | 16 | 17.8 |
| | LRYIAGHFLNFLFYF | 17 | 27.7 |
| 310 | SEGYYKALSNKSTYR | 2 | 4.7 |
| | EGYYKALSNKSTYRH | 3 | 4.7 |
| | GYYKALSNKSTYRHA | 4 | 5 |
| | YYKALSNKSTYRHAL | 5 | 5.6 |
| | LSEGYYKALSNKSTY | 1 | 5.7 |
| | KSTYRHALLSSIFRL | 12 | 6.3 |
| | STYRHALLSSIFRLF | 13 | 7.9 |
| | NKSTYRHALLSSIFR | 11 | 8 |
| | YKALSNKSTYRHALL | 6 | 8.1 |
| | LLSEGYYKALSNKST | 0 | 8.3 |
| | IFRLFLFKLVSMTAK | 23 | 8.8 |
| | TYRHALLSSIFRLFL | 14 | 10.5 |
| | SNKSTYRHALLSSIF | 10 | 10.9 |
| | SIFRLFLFKLVSMTA | 22 | 10.9 |
| | YRHALLSSIFRLFLF | 15 | 15.9 |
| | LSNKSTYRHALLSSI | 9 | 18.5 |
| 349 | LLSFTLLRLVYVAFL | 21 | 12.8 |
| | LLLSFTLLRLVYVAF | 20 | 15.1 |
| | LSFTLLRLVYVAFLV | 22 | 18 |

| | | | |
|---|---|---|---|
| | TLLLSFTLLRLVYVA | 19 | 18.7 |
| | LTLLLSFTLLRLVYV | 18 | 22.1 |
| | ILSLSAIPGSVLSSR | 0 | 5.4 |
| | LSLSAIPGSVLSSRL | 1 | 9.8 |
| | SLSAIPGSVLSSRLM | 2 | 16.8 |
| 355 | LSAIPGSVLSSRLMI | 3 | 28.8 |
| | HRRCLLFLLKFTGGL | 22 | 45.1 |
| | SRLMIKNVAHRRCLL | 13 | 45.7 |
| | GSPYRYMFLKQLNDQ | 2 | 8.9 |
| | SSGSPYRYMFLKQLN | 0 | 10.1 |
| | SGSPYRYMFLKQLND | 1 | 10.5 |
| 363 | SPYRYMFLKQLNDQR | 3 | 11.1 |
| | PYRYMFLKQLNDQRT | 4 | 11.9 |
| | YRYMFLKQLNDQRTG | 5 | 16.1 |
| | RYMFLKQLNDQRTGN | 6 | 31 |
| | RSRYRKRLALRAIGR | 22 | 9.8 |
| | SRSRYRKRLALRAIG | 21 | 12 |
| | FAFLVVKQYLARSNC | 0 | 12.1 |
| | SSRSRYRKRLALRAI | 20 | 14 |
| | DSSRSRYRKRLALRA | 19 | 16.9 |
| | VVKQYLARSNCKIIA | 4 | 18.3 |
| 371 | VKQYLARSNCKIIAD | 5 | 18.4 |
| | AFLVVKQYLARSNCK | 1 | 19.2 |
| | FLVVKQYLARSNCKI | 2 | 20.9 |
| | LVVKQYLARSNCKII | 3 | 24.3 |
| | KQYLARSNCKIIADS | 6 | 26.7 |
| | QYLARSNCKIIADSS | 7 | 46.6 |
| | GFYLSASAQNGRLSI | 0 | 5 |
| | FYLSASAQNGRLSIN | 1 | 5.8 |
| | YLSASAQNGRLSINA | 2 | 8.8 |
| | QNGRLSINAHLVNHF | 8 | 11.2 |
| | AQNGRLSINAHLVNH | 7 | 14.4 |
| | NGRLSINAHLVNHFT | 9 | 16.3 |
| 373 | SAQNGRLSINAHLVN | 6 | 19.4 |
| | GRLSINAHLVNHFTL | 10 | 20.3 |
| | HLVNHFTLQLSIMMS | 17 | 25.2 |
| | RLSINAHLVNHFTLQ | 11 | 25.8 |
| | ASAQNGRLSINAHLV | 5 | 25.8 |
| | VNHFTLQLSIMMSDQ | 19 | 26.6 |

| | | | |
|---|---|---|---|
| | LVNHFTLQLSIMMSD | 18 | 27.2 |
| | NHFTLQLSIMMSDQN | 20 | 36.8 |
| | LSINAHLVNHFTLQL | 12 | 38.4 |
| | AHLVNHFTLQLSIMM | 16 | 42.9 |
| 376 | DFLVTMLINKRACTL | 13 | 5.6 |
| | FLVTMLINKRACTLL | 14 | 6.1 |
| | IDFLVTMLINKRACT | 12 | 7.3 |
| | LVTMLINKRACTLLT | 15 | 9 |
| | RIDFLVTMLINKRAC | 11 | 9.8 |
| | GRIDFLVTMLINKRA | 10 | 11.9 |
| | VTMLINKRACTLLTS | 16 | 16 |
| | NKRACTLLTSVFIGE | 21 | 35.2 |
| | INKRACTLLTSVFIG | 20 | 37.6 |
| | LINKRACTLLTSVFI | 19 | 40.7 |
| 385 | AAMFTSNSPQATLFR | 3 | 7.2 |
| | AMFTSNSPQATLFRW | 4 | 9.8 |
| | TAAMFTSNSPQATLF | 2 | 11.5 |
| | MFTSNSPQATLFRWP | 5 | 14.7 |
| | RWPFLIMPVTVFPST | 17 | 24.9 |
| | TLFRWPFLIMPVTVF | 14 | 25.8 |
| | TTAAMFTSNSPQATL | 1 | 26.5 |
| | FRWPFLIMPVTVFPS | 16 | 29.2 |
| | FTSNSPQATLFRWPF | 6 | 30.5 |
| | LFRWPFLIMPVTVFP | 15 | 30.7 |
| | PFLIMPVTVFPSTYL | 19 | 40.8 |
| | WPFLIMPVTVFPSTY | 18 | 41.8 |
| | FLIMPVTVFPSTYLR | 20 | 44.5 |
| 424 | FTQLRYTVARFNYTT | 19 | 21.8 |
| | CVLFTQLRYTVARFN | 16 | 23.8 |
| | CCVLFTQLRYTVARF | 15 | 25.5 |
| | VLFTQLRYTVARFNY | 17 | 25.7 |
| | LFTQLRYTVARFNYT | 18 | 25.8 |
| | CCCVLFTQLRYTVAR | 14 | 26.7 |
| | ICCCVLFTQLRYTVA | 13 | 33 |
| | QLRYTVARFNYTTGM | 21 | 39 |
| | TQLRYTVARFNYTTG | 20 | 40.5 |
| 433 | EYRTYMYYLVTSYHF | 21 | 8.1 |
| | TVWLLSQQSKCAREY | 8 | 8.3 |
| | VWLLSQQSKCAREYR | 9 | 11.8 |

| | | | |
|---|---|---|---|
| | STVWLLSQQSKCARE | 7 | 15.2 |
| | TSTVWLLSQQSKCAR | 6 | 18.9 |
| | WLLSQQSKCAREYRT | 10 | 21.4 |
| | CTSTVWLLSQQSKCA | 5 | 30.9 |
| | LLSQQSKCAREYRTY | 11 | 43.8 |
| 465 | CQNLILCCTSQPMFI | 20 | 16 |
| | ICQNLILCCTSQPMF | 19 | 32 |
| | QLNFLSRFNTLTQII | 5 | 39 |
| | NQLNFLSRFNTLTQI | 4 | 46.2 |
| 495 | ARRIAKFSAPPGLIL | 6 | 6.6 |
| | RRIAKFSAPPGLILK | 7 | 8.5 |
| | RIAKFSAPPGLILKV | 8 | 10.9 |
| | LARRIAKFSAPPGLI | 5 | 11.3 |
| | IAKFSAPPGLILKVS | 9 | 18.4 |
| | VLARRIAKFSAPPGL | 4 | 22.8 |
| | PGLILKVSSFALISL | 16 | 25.8 |
| | PPGLILKVSSFALIS | 15 | 31.2 |
| | GLILKVSSFALISLL | 17 | 35.4 |
| | APPGLILKVSSFALI | 14 | 37.6 |
| | LKVSSFALISLLQSV | 20 | 49.5 |
| 516 | SVVILAEPASFSLRF | 13 | 5.9 |
| | LSVVILAEPASFSLR | 12 | 6.4 |
| | LLSVVILAEPASFSL | 11 | 7.1 |
| | VVILAEPASFSLRFY | 14 | 7.7 |
| | YLLSVVILAEPASFS | 10 | 10.2 |
| | VILAEPASFSLRFYS | 15 | 11.1 |
| | FYLLSVVILAEPASF | 9 | 16.3 |
| | ILAEPASFSLRFYSH | 16 | 17.7 |
| | FKKRFYLLSVVILAE | 5 | 20.9 |
| | LFKKRFYLLSVVILA | 4 | 21.8 |
| | KRFYLLSVVILAEPA | 7 | 22.1 |
| | RFYLLSVVILAEPAS | 8 | 22.6 |
| | SLFKKRFYLLSVVIL | 3 | 26.7 |
| | KKRFYLLSVVILAEP | 6 | 28.3 |
| 536 | LAAFFLFSNALPYFK | 12 | 4.7 |
| | AAFFLFSNALPYFKP | 13 | 4.8 |
| | SLAAFFLFSNALPYF | 11 | 5.5 |
| | AFFLFSNALPYFKPQ | 14 | 5.6 |
| | FFLFSNALPYFKPQT | 15 | 7 |

| | | | |
|---|---|---|---|
| | FSLAAFFLFSNALPY | 10 | 7.3 |
| | RETLLQAANFSLAAF | 1 | 9.7 |
| | FRETLLQAANFSLAA | 0 | 9.9 |
| | FLFSNALPYFKPQTK | 16 | 12.2 |
| | ETLLQAANFSLAAFF | 2 | 14.4 |
| | TLLQAANFSLAAFFL | 3 | 18.5 |
| | NFSLAAFFLFSNALP | 9 | 25.9 |
| | LLQAANFSLAAFFLF | 4 | 33.7 |
| 539 | HLHVILLSSENLPIS | 7 | 7.2 |
| | HVILLSSENLPISLI | 9 | 7.6 |
| | LHVILLSSENLPISL | 8 | 7.7 |
| | PHLHVILLSSENLPI | 6 | 8.7 |
| | VILLSSENLPISLIF | 10 | 10.5 |
| | ITPHLHVILLSSENL | 4 | 11.1 |
| | YRTIITPHLHVILLS | 0 | 11.1 |
| | TPHLHVILLSSENLP | 5 | 12.2 |
| | ILLSSENLPISLIFK | 11 | 19.1 |
| | LLSSENLPISLIFKL | 12 | 37.3 |
| 619 | TFLLLIVKSRIIKSK | 6 | 6.4 |
| | FTFLLLIVKSRIIKS | 5 | 7.1 |
| | CFTFLLLIVKSRIIK | 4 | 8 |
| | FLLLIVKSRIIKSKG | 7 | 8.4 |
| | RCFTFLLLIVKSRII | 3 | 9.8 |
| | KSKGSFNEMLTMRSV | 18 | 10.6 |
| | LLLIVKSRIIKSKGS | 8 | 12.8 |
| | IKSKGSFNEMLTMRS | 17 | 17 |
| | LLIVKSRIIKSKGSF | 9 | 21.2 |
| | KSRIIKSKGSFNEML | 13 | 28.7 |
| | IVKSRIIKSKGSFNE | 11 | 47.4 |
| 623 | NLNIKQLMTQEIRLK | 8 | 7.7 |
| | LNIKQLMTQEIRLKI | 9 | 9.6 |
| | NNLNIKQLMTQEIRL | 7 | 10.7 |
| | NIKQLMTQEIRLKIF | 10 | 18.5 |
| | ENNLNIKQLMTQEIR | 6 | 23.8 |
| | IKQLMTQEIRLKIFL | 11 | 28.8 |
| 629 | PALFIFCLIAAFLLR | 5 | 11.7 |
| | ALFIFCLIAAFLLRL | 6 | 11.9 |
| | LFIFCLIAAFLLRLH | 7 | 13.3 |
| | IPALFIFCLIAAFLL | 4 | 14.7 |

| | | | |
|---|---|---|---|
| | FIFCLIAAFLLRLHD | 8 | 21.4 |
| | IFCLIAAFLLRLHDW | 9 | 36.3 |
| | AIPALFIFCLIAAFL | 3 | 40.8 |
| 778 | LPFLARINAQLTYQS | 11 | 4.6 |
| | FLPFLARINAQLTYQ | 10 | 5 |
| | SFLPFLARINAQLTY | 9 | 5.6 |
| | PFLARINAQLTYQSI | 12 | 5.8 |
| | FLARINAQLTYQSIA | 13 | 6.8 |
| | FSFLPFLARINAQLT | 8 | 7.5 |
| | LARINAQLTYQSIAE | 14 | 11.5 |
| | PFSFLPFLARINAQL | 7 | 28.8 |
| 787 | KKTYEILAGLKRHTT | 11 | 10.1 |
| | IKKTYEILAGLKRHT | 10 | 12.1 |
| | SIKKTYEILAGLKRH | 9 | 15.1 |
| | KTYEILAGLKRHTTV | 12 | 16.8 |
| | QSIKKTYEILAGLKR | 8 | 19.9 |
| | TYEILAGLKRHTTVF | 13 | 27.7 |
| | YEILAGLKRHTTVFM | 14 | 29.5 |
| 804 | RGFYLTLLPETALIS | 9 | 4 |
| | RRGFYLTLLPETALI | 8 | 4.2 |
| | GRRGFYLTLLPETAL | 7 | 4.4 |
| | GFYLTLLPETALISR | 10 | 4.6 |
| | NGRRGFYLTLLPETA | 6 | 5 |
| | FYLTLLPETALISRS | 11 | 5.3 |
| | YLTLLPETALISRSS | 12 | 6.6 |
| | LLPETALISRSSKLI | 15 | 32.7 |
| | LTLLPETALISRSSK | 13 | 36.8 |
| 833 | SGAFFSARAFFVSYL | 4 | 6.9 |
| | ESGAFFSARAFFVSY | 3 | 7.9 |
| | GAFFSARAFFVSYLY | 5 | 9 |
| | HESGAFFSARAFFVS | 2 | 9.4 |
| | LHESGAFFSARAFFV | 1 | 10.9 |
| | AFFSARAFFVSYLYF | 6 | 12.7 |
| | VSYLYFKVTAKTDST | 15 | 13.2 |
| | FFSARAFFVSYLYFK | 7 | 19.1 |
| | FVSYLYFKVTAKTDS | 14 | 21.3 |
| | FFVSYLYFKVTAKTD | 13 | 38.2 |
| 905 | VFHYRFLFSRFTDYR | 13 | 4.4 |
| | TVFHYRFLFSRFTDY | 12 | 4.7 |

| | | | |
|---|---|---|---|
| | FHYRFLFSRFTDYRR | 14 | 4.9 |
| | NETVFHYRFLFSRFT | 10 | 5.1 |
| | ETVFHYRFLFSRFTD | 11 | 5.1 |
| | HYRFLFSRFTDYRRL | 15 | 5.9 |

### Table S3: Pairwise Sequence Comparison between *Synpeps* and Amyloid $beta_{42}$ peptide sequence

| Synpep ID | Identity (%) | Query region aligned | Subject region aligned |
|---|---|---|---|
| PSP86 | 23 | 4 to 16 | 21 to33 |
| PSP98 | 22 and 50 | 14 to 31 & 36 to 39 | 30 to 47 & 44 to 47 |
| PSP193 | 43 | 8 to 14 | 8 to 14 |
| PSP226 | 57 | 14 to20 | 34 to 40 |
| PSP461 | 100 | 14 to 15 | 23 to 24 |
| PSP536 | 100 | 19 to 20 | 16 to 17 |
| PSP793 | 27 | 4 to 18 | 8 to 22 |
| PSP846 | 56 | 1 to 9 | 16 to 24 |

### Table S4: B cell epitope prediction for toxic Amyloid $Beta_{42}$ peptide

| B cell epitopes of Amyloid beta peptide | | | | | | | |
|---|---|---|---|---|---|---|---|
| Linear | Start | End | Number | Score | Discontinuous | Number | Score |
| VGGVVIA | 36 | 42 | 7 | 0.765 | D1, A2, E3, F4, R5 | 5 | 0.852 |
| DAEFRHDS | 1 | 8 | 8 | 0.738 | N27, G29, A30, I32, V36, G37, G38, V39, V40, I41, A42 | 11 | 0.669 |
| SNKGA | 26 | 30 | 5 | 0.586 | | | |

### Table S5: Physico-chemical properties and predicted homologs of selected mimotopes and $A\beta_{42}$ epitope

| Epitope ID | Molecular weight (Da) | Isoelectric point | Homologue in Blast P search (Acc. No) | Homologue description | E value | Query coverag e (%) |
|---|---|---|---|---|---|---|

| | | | | | | |
|---|---|---|---|---|---|---|
| Epi_Abeta$_{42}$ | 1196.2 | 5.59 | 2IPU_P | Chain P, Pfa1 Fab Fragment Complexed With Abeta 1-8 Peptide [synthetic construct] | 3.3 | 100 |
| Mimo_ PSP172 | 1018.1 | 5.776 | CCK73009.1 | Hypothetical protein KNAG_0M01560 [*Kazachstania naganishii* CBS 8797] | 64 | 100 |
| Mimo_ PSP264 | 1347.6 | 7.769 | WP_032431 326.1 | Hypothetical protein [*Klebsiella pneumoniae*] | 5.2 | 100 |
| Mimo_ PSP572 | 1105.2 | 5.986 | XP_0078045 05.1 | Hypothetical protein EPUS_09056 [*Endocarpon pusillum* Z07020] | 116 | 87 |
| Mimo_ PSP629 | 1087.3 | 5.954 | CBA10082.1 | Polyribonucleotide nucleotidyltransfera se [*Neisseria meningitidis* alpha275] | 480 | 87 |
| Mimo_ PSP776 | 1142.4 | 6.199 | WP_029460 120.1 | Hypothetical protein [*Desulfovibrio alcoholivorans*] | 62 | 100 |
| Mimo_ PSP778 | 979 | 5.846 | EMT03525.1 | Hypothetical protein F775_12298 [*Aegilops tauschii*] | 83 | 100 |
| Mimo_ PSP623 | 1070.1 | 5.827 | KFP87772.1 | TOM1-like 1 [*Apaloderma vittatum*] | 399 | 100 |

**Table S6: Scores of mimotope modeling from ITASSER ; RMSD[a] between the structurally aligned regions of the query with templates used in ITASSER; RMSD[b] for structural superimposition of predicted models (Itasser/ pepfold) with native epitope of Aβ$_{42}$ (4ONF)**

| **Mimotope Id** | **C-Score (Itasser)** | **RMSD[a] ($A^0$)** | **RMSD[b] ($A^0$) -Native ligand** |
|---|---|---|---|
| Mimo_PSP172 | -0.44 | 0.6±0.6 | 0.59/0.44 |
| Mimo_PSP264 | -0.04 | 0.5±0.5 | 0.52/0.56 |

| | | | |
|---|---|---|---|
| Mimo_PSP572 | -0.79 | 1.2±1.2 | 2.66/0.80 |
| Mimo_PSP623 | -1.05 | 1.6±1.4 | 2.13/2.26 |
| Mimo_PSP629 | -0.02 | 0.5±0.5 | 0.62/0.49 |
| Mimo_PSP776 | -0.09 | 0.5±0.5 | 2.05/2.57 |
| Mimo_PSP778 | -0.89 | 1.4±1.3 | 2.84/3.19 |

**Table S7: Molecular interaction between Fab and our 7 lead mimotopes - (WHB : Water Hydrogen Bond, CHB : Carbon Hydrogen Bond, CoHB : Conventional Hydrogen Bond, Pi-DHB: Pi-Donor Hydrogen Bond)**

| Ligand | ZDOCK score | ZRANK score | Favorable Interaction | Interaction Type | Bond distances |
|---|---|---|---|---|---|
| Native ligand (Abeta epitope) | 11.52 | -86.231 | L:ARG101:NH2 - P:GLU3:OE2 | Salt Bridge | 2.20 |
| | | | P:ARG5:NH2 - L:ASP31:OD1 | Salt Bridge | 2.95 |
| | | | L:ARG101:NH2 - P:ASP1:OD1 | Attractive Charge | 4.21 |
| | | | P:ARG5:NH1 - L:ASP31:OD2 | Attractive Charge | 3.52 |
| | | | P:ARG5:NH1 - L:ASP33:OD2 | Attractive Charge | 4.01 |
| | | | P:HOH103:O - P:PHE4:O | WHB;CoHB | 2.97 |
| | | | P:HOH104:O - P:ALA2:O | WHB;CoHB | 3.39 |
| | | | P:HOH107:O - P:HIS6:O | WHB;CoHB | 2.33 |
| | | | H:SER50:OG - P:GLU3:OE2 | CoHB | 3.39 |
| | | | H:ARG52:NE - P:GLU3:O | CoHB | 3.36 |
| | | | H:ARG57:NH2 - P:HIS6:O | CoHB | 3.17 |
| | | | H:SER106:N - P:ASP1:OD2 | CoHB | 3.27 |
| | | | H:SER106:OG - P:ASP1:OD1 | CoHB | 2.97 |
| | | | P:ARG5:NH2 - L:GLY96:O | CoHB | 3.00 |
| | | | P:ARG5:NH2 - L:TYR37 | Pi-Cation | 3.93 |
| | | | P:GLU3:OE2 - H:TRP47 | Pi-Anion | 3.84 |
| | | | H:SER50:OG - P:PHE4 | Pi-DHB | 4.07 |
| | | | H:TYR99 - P:PHE4 | Pi-Pi Stacked | 4.38 |
| PSP-172 | 9.16 | -78.299 | P:HOH103:O - :ASN9:O | WHB;CoHB | 2.97 |
| | | | :ASN9:H - P:HOH103:O | WHB;CoHB | 2.37 |
| | | | H:ARG52:NH1 - :ASN9:O | CoHB | 2.68 |
| | | | H:TYR99:OH - :THR3:O | CoHB | 2.17 |

| | | | | | |
|---|---|---|---|---|---|
| | | | :CYS1:H1 - L:THR97:O | CoHB | 2.81 |
| | | | :CYS1:HG - L:GLN95:OE1 | CoHB | 2.00 |
| | | | :SER10:H - H:SER53:OG | CoHB | 2.73 |
| | | | H:GLY33:CA - :ILE8:O | CHB | 3.02 |
| | | | H:ARG52:CA - :ILE8:O | CHB | 2.31 |
| | | | H:ARG52:CD - :ASN9:O | CHB | 3.46 |
| | | | H:GLY54:CA - :SER10:OG | CHB | 3.41 |
| | | | :PRO4:CD - L:GLY96:O | CHB | 2.48 |
| | | | :LEU2:CD1 - H:TRP47 | Pi-Sigma | 3.18 |
| | | | L:ARG101 - :LEU2 | Alkyl | 4.69 |
| | | | H:TRP47 - :LEU2 | Pi-Alkyl | 5.17 |
| | | | H:TYR99 - :ALA5 | Pi-Alkyl | 4.53 |
| | | | H:TYR99 - :ILE8 | Pi-Alkyl | 3.63 |
| | | | H:HIS101 - :ALA5 | Pi-Alkyl | 5.22 |
| | | | L:TYR37 - :PRO4 | Pi-Alkyl | 3.55 |
| PSP-264 | 8.44 | -49.119 | :ILE1:H2 - L:ASP33:OD2 | Salt Bridge;AC | 2.40 |
| | | | :ARG8:HH21 - L:ASP31:OD1 | Salt Bridge;AC | 2.31 |
| | | | :ARG8:NH1 - L:ASP31:OD2 | Attractive Charge | 4.79 |
| | | | P:HOH105:O - :VAL4:O | WHB;CoHB | 3.19 |
| | | | P:HOH105:O - :MET7:SD | WHB;CoHB | 3.71 |
| | | | :HIS5:HE2 - P:HOH101:O | WHB;CoHB | 2.15 |
| | | | :ARG8:H - P:HOH104:O | WHB;CoHB | 1.97 |
| | | | :HIS9:HE1 - P:HOH101:O | WHB;CoHB | 2.74 |
| | | | :ARG10:HE - P:HOH102:O | WHB;CoHB | 2.18 |
| | | | L:TYR37:OH - :ILE1:O | CoHB | 3.24 |
| | | | :ARG8:HH21 - L:THR97:O | CoHB | 2.80 |
| | | | :ARG2:NH2 - H:TYR102 | Pi-Cation | 4.01 |
| | | | :ARG8:NH2 - L:HIS98 | Pi-Cation | 4.86 |
| | | | :ARG10:NH2 - H:TYR59 | Pi-Cation | 2.87 |
| | | | H:TYR99 - :LYS6 | Pi-Alkyl | 4.57 |
| | | | H:HIS101 - :ARG2 | Pi-Alkyl | 5.44 |
| | | | :HIS9 - L:ARG101 | Pi-Alkyl | 3.46 |
| | | | P:HOH102:O - :HIS9:O | WMHB;CoHB | 1.78 |
| | | | P:HOH104:O - :VAL4:O | WHB;CoHB | 1.82 |
| | | | P:HOH106:O - L:ASP31:OD1 | WHB;CoHB | 2.73 |
| | | | :ARG8:CD - L:THR97:O | CHB | 2.14 |

| | | | | | |
|---|---|---|---|---|---|
| PSP-572 | 7.42 | -92.014 | H:ARG52:NH1 - :ASP10:OD2<br>:CYS1:N - L:ASP33:OD2<br>P:HOH103:O - :ASP10:OD2<br>P:HOH104:O - :GLN5:OE1<br>P:HOH105:O - :GLN5:OE1<br>P:HOH106:O - :GLN5:OE1<br>:CYS1:H2 - P:HOH104:O<br>:CYS1:H3 - P:HOH104:O<br>H:MET34:N - :VAL7:O<br>H:SER53:N - :ARG8:O<br>:CYS1:HG - L:THR97:OG1<br>:SER2:H - L:THR97:O<br>:ARG8:HH11 - H:TYR99:OH<br>:CYS1:CA - L:THR97:O<br>:CYS1:N - L:TYR37<br>L:ARG101 - :PRO3<br>H:TYR99 - :LEU4<br>L:PHE99 - :MET6<br>:CYS1:H1 - L:ASP31:OD1<br>L:ARG101:N - P:HOH102:O<br>P:HOH102:O - H:TRP47<br>P:HOH102:O - H:TRP47<br>P:HOH106:O - L:ASP31:OD1<br>:CYS1:H2 - P:HOH106:O<br>L:GLY96:CA - P:HOH101:O<br>P:HOH103:O - :GLY9:O<br>:SER2:H - L:THR97:O | Attractive Charge<br>Attractive Charge<br>WHB;CoHB<br>WHB;CoHB<br>WHB;CoHB<br>WHB;CoHB<br>WHB;CoHB<br>WHB;CoHB<br>CoHB<br>CoHB<br>CoHB<br>CoHB<br>CoHB<br>CHB<br>Pi-Cation<br>Alkyl<br>Pi-Alkyl<br>Pi-Alkyl<br>Salt Bridge<br>WMHB;CHB<br>WMHB;Pi-DHB<br>WMHB;Pi-DHB<br>WMHB;CoHB<br>WMHB;CoHB<br>WHB;CHB<br>CoHB | 4.34<br>5.32<br>2.93<br>2.17<br>3.13<br>2.62<br>2.62<br>2.84<br>2.62<br>2.40<br>2.97<br>2.48<br>1.59<br>2.60<br>4.95<br>4.16<br>4.82<br>5.01<br>1.71<br>2.92<br>3.58<br>3.30<br>2.73<br>1.58<br>3.36<br>1.76<br>2.48 |
| PSP-623 | 9.80 | -71.723 | L:ARG101:NH2 - :GLU7:OE1<br>:ARG5:HH11 - L:ASP31:OD1<br>H:ARG52:NH2 - :GLU6:OE2<br>H:ARG57:NH2 - :GLU6:OE2<br>:ARG5:NH1 - L:ASP33:OD2<br>P:HOH104:O - :ASN9:OD1<br>P:HOH105:O - :ASN9:OD1<br>:ARG5:HE - P:HOH104:O<br>:ARG5:HE - P:HOH106:O<br>:ARG5:HH11 - P:HOH106:O<br>H:SER50:OG - :GLU7:OE1<br>H:TYR99:OH - :ASN9:O1<br>:ARG5:HH12 - L:GLY96:O<br>:ASN9:H - H:TYR99:OH | Salt Bridge<br>Salt Bridge;AC<br>AC<br>AC<br>AC<br>WHB;CoHB<br>WHB;CoHB<br>WHB;CoHB<br>WHB;CoHB<br>WHB;CoHB<br>CoHB<br>CoHB<br>CoHB<br>CoHB | 3.8538<br>2.74192<br>5.40697<br>5.5776<br>5.25554<br>2.8919<br>2.87436<br>2.08552<br>1.61652<br>2.82343<br>2.57307<br>3.12438<br>2.75071<br>2.64909 |

| | | | | | |
|---|---|---|---|---|---|
| | | | :ASN9:HD21 - L:TYR37:OH<br>:ASN9:HD22 - L:ASP31:OD2<br>:ASN9:HD22 - L:ASP33:OD2<br>:ARG5:NH1 - L:TYR37<br>:GLU6:C,O;GLU7:N - H:TYR59<br>L:PHE99 - :ALA2 | CoHB<br>CoHB<br>CoHB<br>Pi-Cation<br>Amide-Pi Stacked<br>Pi-Alkyl | 2.55663<br>2.97176<br>2.16372<br>3.80094<br>5.43583<br>4.1536 |
| PSP-629 | 9.84 | -86.392 | P:HOH101:O - :PHE2<br>P:HOH102:O - :VAL1:O<br>:VAL1:H3 - P:HOH102:O<br>:ALA4:H - P:HOH103:O<br>L:ASP31:OD1 - :PHE9<br>:ALA7:CB - L:PHE99<br>H:TYR99 - :PHE2<br>H:TRP47 - :VAL1<br>H:TRP47 - :VAL1<br>H:TYR59 - :VAL1<br>H:TYR59 - :PRO6<br>H:TYR59 - :ALA7<br>H:TYR99 - :PRO3<br>H:TYR99 - :ALA4 | WHB;Pi-DHB<br>WHB;CoHB<br>WHB;CoHB<br>WHB;CoHB<br>Pi-Anion<br>Pi-Sigma<br>Pi-Pi Stacked<br>Pi-Alkyl<br>Pi-Alkyl<br>Pi-Alkyl<br>Pi-Alkyl<br>Pi-Alkyl<br>Pi-Alkyl<br>Pi-Alkyl | 3.61481<br>3.29093<br>2.50283<br>2.70353<br>4.10767<br>2.77316<br>5.01329<br>5.49406<br>4.77238<br>5.22651<br>5.37674<br>5.01039<br>4.48472<br>5.39923 |
| PSP-776 | 10.14 | -64.491 | P:HOH103:O - :GLN4:OE1<br>:ILE7:CA - P:HOH105:O<br>:GLN8:H - P:HOH104:O<br>:GLN8:H - P:HOH105:O<br>H:ARG52:NE - :GLN4:O<br>L:ARG101:NE - :LEU1:O<br>:LEU1:H1 - L:GLN95:O<br>:LEU1:H2 - L:GLN95:O<br>:GLN4:HE22 - H:SER53:OG<br>H:ARG52:CD - :PRO3:O<br>:PRO3:CD - H:SER106:OG<br>L:ARG101 - :LEU1<br>H:TYR59 - :CYS6<br>H:TYR99 - :PRO3<br>H:TYR99 - :VAL5<br>L:PHE99 - :LEU1 | WHB;CoHB<br>WHB;CoHB<br>WHB;CoHB<br>WHB;CoHB<br>CoHB<br>CoHB<br>CoHB<br>CoHB<br>CoHB<br>CHB<br>CHB<br>Alkyl<br>Pi-Alkyl<br>Pi-Alkyl<br>Pi-Alkyl<br>Pi-Alkyl | 2.13307<br>3.63673<br>2.83181<br>2.70974<br>3.03991<br>2.47968<br>2.34131<br>2.49439<br>2.44933<br>3.40326<br>3.51446<br>4.70869<br>4.89806<br>5.3929<br>5.05675<br>5.33155 |
| PSP-778 | 8.36 | -79.553 | :SER1:HG - P:HOH103:O<br>:SER2:CB - P:HOH106:O<br>:PRO3:CD - P:HOH106:O<br>:SER4:HG - P:HOH106:O<br>L:GLY96:CA - :PRO8:O<br>L:ARG101:NE - :PHE9<br>H:TRP47 - :PHE9 | WHB;CoHB<br>WHB;CHB<br>WHB;CHB<br>WHB;CoHB<br>CHB<br>Pi-DHB<br>Pi-Pi T-shaped | 2.35<br>3.55<br>3.48<br>2.72<br>3<br>3.47<br>4.25 |

| | | | | | |
|---|---|---|---|---|---|
| | | | H:TRP47 - :PHE9<br>L:PHE99 - :PHE9<br>:LEU7:C,O;PRO8:N-H:TYR99<br>H:TYR59 - :LEU7<br>H:TYR99 - :PRO8 | Pi-Pi T-shaped<br>Pi-Pi T-shaped<br>Amide-Pi Stacked<br>Pi-Alkyl<br>Pi-Alkyl | 4.51<br>5.37<br>3.66<br>5.06<br>4.33 |

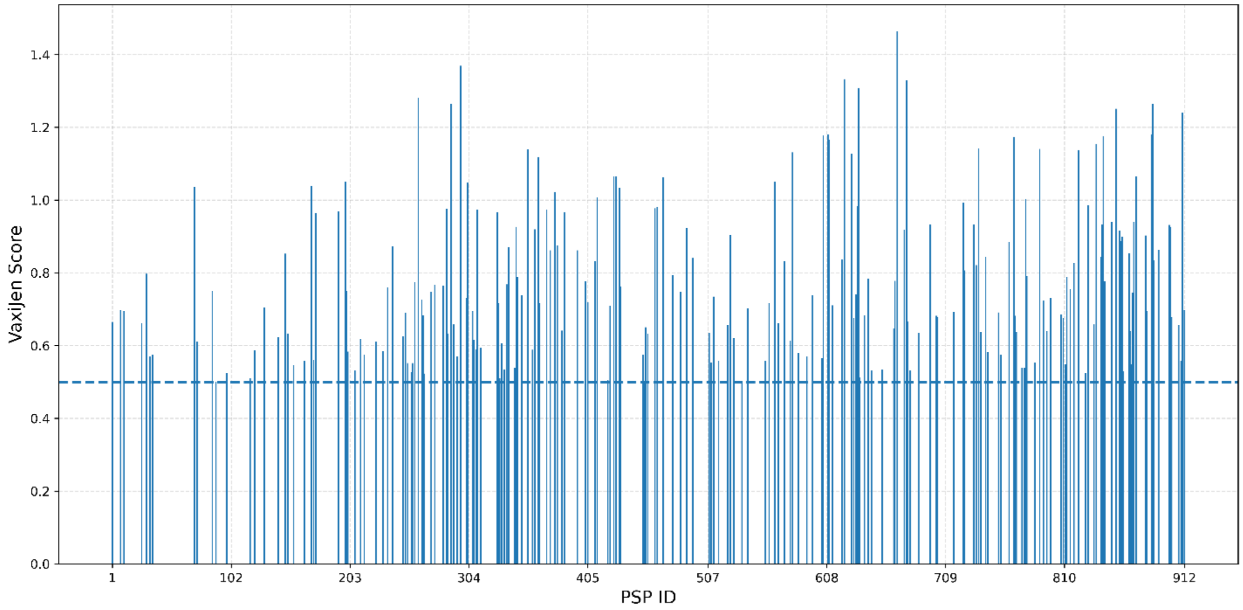


**Figure S1. Distribution of VaxiJen antigenicity scores across Synpep PSP IDs.**
Scatter plot showing VaxiJen scores for individual Synpeps indexed by PSP ID. The dashed horizontal line indicates the antigenicity threshold (VaxiJen score = 0.5), above which peptides are considered predicted antigens. Synpeps exceeding this cutoff represent putative antigenic candidates prioritized for downstream epitope binding and immunogenicity analyses.

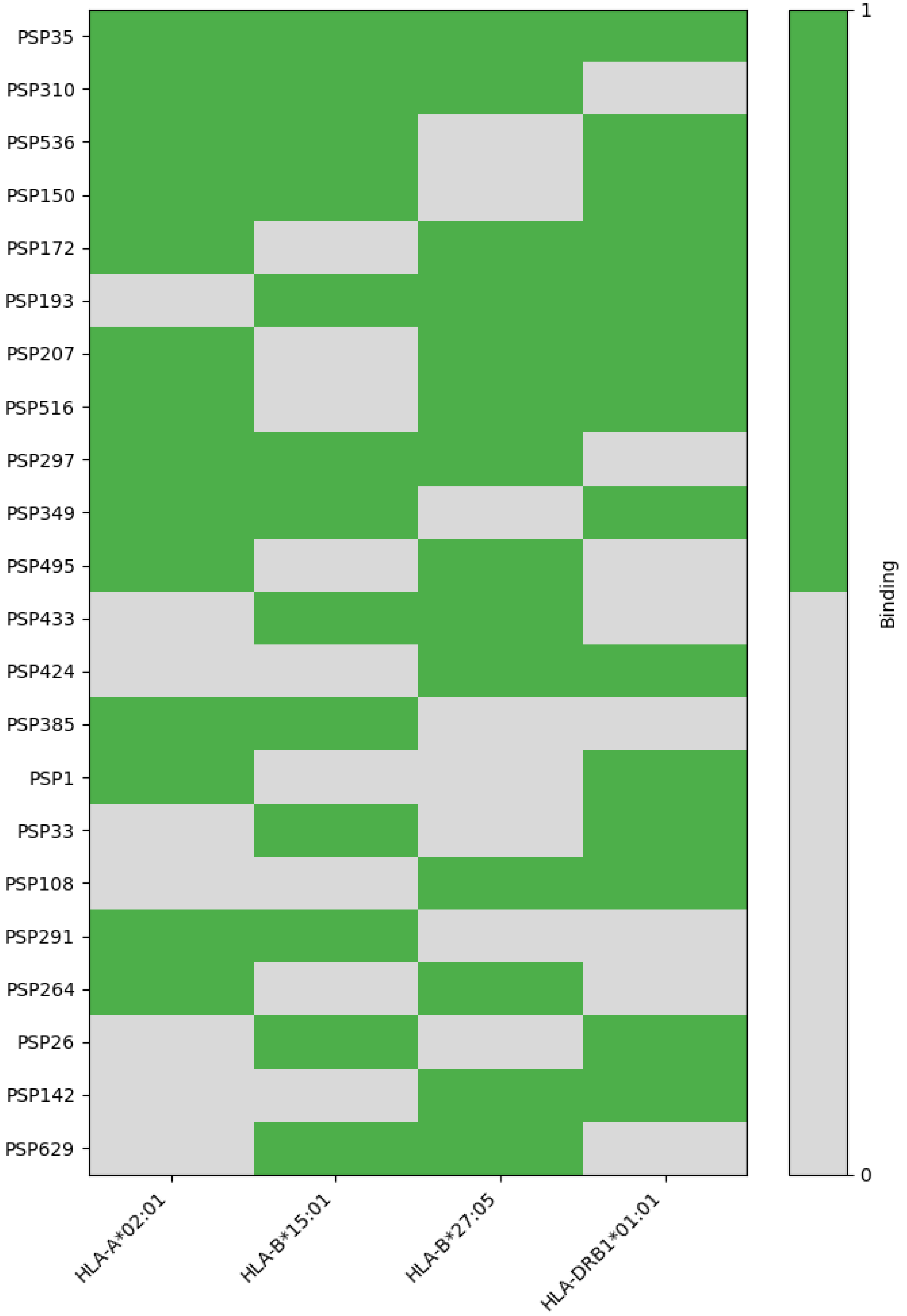

PSP35
PSP310
PSP536
PSP150
PSP172
PSP193
PSP207
PSP516
PSP297
PSP349
PSP495
PSP433
PSP424
PSP385
PSP1
PSP33
PSP108
PSP291
PSP264
PSP26
PSP142
PSP629
HLA-A*02:01
HLA-B*15:01
HLA-B*27:05
HLA-DRB1*01:01
1
0
Binding

Figure S2. Binding profile of Synpep epitopes across MHC class I and II alleles.

Heatmap depicting the binding profile of the top 50 Synpep epitopes across HLA-A02:01, HLA-B15:01, HLA-B27:05, and HLA-DRB101:01. Binding interactions are indicated in green, while grey denotes lack of predicted binding. Synpeps are labeled by PSP ID. The figure highlights allele-specific binding patterns and limited cross-allelic promiscuity between MHC class I and class II molecules.
